# A HIF1a-Dependent Pro-Oxidant State Disrupts Synaptic Plasticity and Impairs Spatial Memory in Response to Intermittent Hypoxia

**DOI:** 10.1523/ENEURO.0024-20.2020

**Published:** 2020-06-19

**Authors:** Alejandra Arias-Cavieres, Maggie A. Khuu, Chinwendu U. Nwakudu, Jasmine E. Barnard, Gokhan Dalgin, Alfredo J. Garcia

**Affiliations:** 1 Institute for Integrative Physiology, The University of Chicago, Chicago, IL 60637; 2 Grossman Institute for Neuroscience, Quantitative Biology and Human Behavior, The University of Chicago, Chicago, IL 60637; 3 Department of Medicine, Section of Emergency Medicine, The University of Chicago, Chicago, IL 60637; 4 Department of Medicine Section of Adult and Pediatric Endocrinology, Diabetes, and Metabolism, The University of Chicago, Chicago, IL 60637

**Keywords:** hypoxia inducible factor, NADPH oxidase, NMDA receptor, oxidative stress, sleep apnea

## Abstract

Sleep apnea causes cognitive deficits and is associated with several neurologic diseases. Intermittent hypoxia (IH) is recognized as a principal mediator of pathophysiology associated with sleep apnea, yet the basis by which IH contributes to impaired cognition remains poorly defined. Using a mouse model exposed to IH, this study examines how the transcription factor, hypoxia inducible factor 1a (HIF1a), contributes to disrupted synaptic physiology and spatial memory. In wild-type mice, impaired performance in the Barnes maze caused by IH coincided with a loss of NMDA receptor (NMDAr)-dependent long-term potentiation (LTP) in area CA1 and increased nuclear HIF1a within the hippocampus. IH-dependent HIF1a signaling caused a two-fold increase in expression of the reactive oxygen species (ROS) generating enzyme NADPH oxidase 4 (NOX4). These changes promoted a pro-oxidant state and the downregulation of GluN1 within the hippocampus. The IH-dependent effects were not present in either mice heterozygous for *Hif1a* (HIF1a^+/−^) or wild-type mice treated with the antioxidant manganese (III) tetrakis(1-methyl-4-pyridyl) porphyrin (MnTMPyP). Our findings indicate that HIF1a-dependent changes in redox state are central to the mechanism by which IH disrupts hippocampal synaptic plasticity and impairs spatial memory. This mechanism may enhance the vulnerability for cognitive deficit and lower the threshold for neurologic diseases associated untreated sleep apnea.

## Significance Statement

Sleep apnea is associated with cognitive decline and neurologic disease. Intermittent hypoxia (IH), a hallmark consequence of sleep apnea, yet the mechanisms by which IH affects cognition is poorly understood. We show that a pro-oxidant state produced by HIF1a is a central factor causing IH-dependent impairment to spatial memory and synaptic plasticity. This work identifies potential targets for intervention in mitigating cognitive decline associated with sleep apnea.

## Introduction

The hippocampus is widely regarded for its importance in learning and memory and is frequently identified as a brain structure impacted by sleep apnea (Sforza et al., 2016; Cha et al., 2017; Macey et al., 2018; Song et al., 2018). As cognitive decline is a recognized comorbidity of sleep apnea (Wallace and Bucks, 2013; Varga et al., 2014; Gildeh et al., 2016; Devita et al., 2017a,b; Leng et al., 2017), changes to hippocampal physiology may have a significant role in disrupting cognition. Intermittent hypoxia (IH) is a hallmark of the sleep apnea and impairs spatial learning and memory (Row et al., 2002; Gozal et al., 2003). These impairments coincide with weakened synaptic plasticity in area CA1 of the hippocampus (Goldbart et al., 2003; Payne et al., 2004; Xie et al., 2010; Zhang et al., 2012; Wall et al., 2014) and the production of oxidative stress in the brain (Nair et al., 2011; Chou et al., 2013). Impaired synaptic plasticity and oxidative stress have been implicated in causing IH-dependent deficits to cognition, but the mechanistic basis by which IH impairs learning and memory remains elusive.

The transcription factor, hypoxia inducible factor 1a (HIF1a) is a critical mediator of cellular adaptations to hypoxia, and is capable of promoting the generation of reactive oxygen species (ROS) that can lead to oxidative stress (Semenza and Prabhakar, 2015). IH increases HIF1a in the hippocampal formation (Chou et al., 2013; Wall et al., 2014). However, the role of IH-dependent HIF1a signaling on changes to the neurophysiological processes underlying cognition remains poorly understood. HIF1a signaling may have an important protective pro-survival role in the brain preserving function in response to the hypoxia experienced during IH. Alternatively, HIF1a may serve as a pro-oxidant transcription factor leading to oxidative stress and impaired neurophysiology. Here, we seek to resolve the role of HIF1a in IH-dependent changes to cognition and the synaptic plasticity. Our experiments demonstrate that enhanced HIF1a signaling promotes a pro-oxidant condition that impairs NMDA receptor (NMDAr)-dependent synaptic plasticity at the local circuit level and contributes deficits in spatial memory.

## Materials and Methods

### Study approval

In accordance with National Institutes of Health guidelines, all animal procedures were performed in accordance with the University of Chicago animal care committee’s regulations.

### Animals

Animals were housed in AAALAC-approved facilities with a 12/12 h light/dark cycle and given *ad libitum* access to food and water. Experiments were performed on wild-type mice and HIF1a^+/−^ (Iyer et al., 1998; Peng et al., 2006) from both sexes (Postnatal day 50 to 80). Unless explicitly stated, no sex-based differences were observed throughout the experiments conducted. All animals were maintained on a C57BL/6 background. Automated genotyping was performed independently by a commercial service (Transnetyx Inc).

### IH exposure

Male and female mice were exposed to chronic IH for 10 consecutive days (IH_10_). In brief, as previously described (Peng and Prabhakar, 2003), the IH_10_ paradigm was performed in a special chamber during the light cycle and lasted 8 h/d (i.e., 80 IH cycles/d). A single hypoxic cycle was achieved by flowing 100% N_2_ into the chamber for ∼60 s (nadir O_2_ reached 4.5 ± 1.5%) and followed immediately by an air break (∼21% O_2_; 300 s).

In a subset of animals used for behavioral experiments, manganese (III) tetrakis(1-methyl-4-pyridyl) porphyrin (MnTMPyP; Enzo Life Sciences, catalog #ALX-430–070) was administered via intraperitoneal injection at the beginning of each day before exposure to IH. Previous reports have indicated that dose of MnTMPyP at either 5 mg/kg (Peng et al., 2006) or 15 mg/kg (Khuu et al., 2019) can mitigate the effects of IH in the nervous system. Therefore, the smaller dose (5 mg/kg, *n* = 9 mice) and the larger dose (15 mg/kg, *n* = 3 mice) were used but no differences were evident between dosage groups; and therefore, the data at the two concentrations were pooled.

### Barnes maze

The Barnes maze was performed using a custom made opaque white circular acrylic platform (92.4 cm in diameter) with 20 equidistant holes (5.08 cm in diameter and 2.54 cm from the edge). The platform was elevated (30 cm from the floor) ground and surrounded by four identical walls (27.94 cm high). By default, each hole was closed with a fixed piece of opaque acrylic that could be removed to lead to a dark exit box. Lighting was achieved through diffuse overhead fluorescent lighting such that all holes were equally lit. An overhead camera was suspended above the maze allowing for video tracking. Data collection and *post hoc* analysis was performed using CinePlex Video Tracking System (Plexon).

As previously described (Christakis et al., 2012), the task was performed using a 4-d protocol consisting of one training trial per day for three consecutive days and a probe trial on the fourth day. Barnes maze began on the seventh day of IH_10_ exposure with respective controls run at the same time. In IH mice, all training trials and the probe trial were conducted before IH exposure on days 7–10. For the training trials, all, but one of the holes (exit hole), were closed. Closed holes were defined as false exits in the training and probe trials. An exit box with a small ramp was placed directly underneath the exit hole. Animals were given a maximum of 6 min to locate the exit. If the mouse found and entered the exit before the 6-min mark, the trial ended. The time of exit was reported as total latency for the trial. If the mouse was unable to locate the exit by 6 min, they were gently guided to the exit and total latency for the trial was reported as 360 s. At end of each trials, the mouse was promptly returned to its home cage. During the probe trial, all holes were closed, and the animal was given 6 min to explore the maze. Latency to initial entry and distance to initial entry into the exit zone were reported. All subjects entered the exit zone during the probe trial. The total number of entries for each false exit and the exit were recorded and used to calculate entry probability.

Entry probability for each false exit and the exit zone during the probe trial was calculated by the following:

$$EP_{n}=100\text{\%}\times \frac{x_{n}}{x_{total}},$$

where *EP_n_*= entry probability for the exit zone; *x_n_*= number of entries into hole n; and *x_total_* = sum of entries into exit zone and false exits.

The entire arena was sanitized in-between trials. Following the end of behavioral testing, IH animals were immediately placed into the IH chamber for exposure.

### Slice preparation

As previously described (Khuu et al., 2019), acute coronal hippocampal slices were prepared from mice unexposed to IH or from mice exposed to IH for 10 d. Tissue harvest occurred within 1–2 d following IH_10_. Mice were anesthetized with isoflurane and euthanized via rapid decapitation. The cerebrum was immediately harvested and blocked, rinsed with cold artificial CSF (aCSF), and mounted for vibratome sectioning. The mounted brain tissue was submerged in aCSF (4°C; equilibrated with 95% O_2_, 5% CO_2_) and coronal cortico-hippocampal brain slices (350 μ;m thick) were prepared. Slices were then immediately transferred into a holding chamber containing aCSF equilibrated with 95% O_2_, 5% CO_2_ (at 20.5 ± 1°C). Slices were allowed to recover for a minimum of one hour before recording and used up to eight hours following tissue harvest. The composition of aCSF was as following: 118 mm NaCl, 10 mm glucose, 20 mm sucrose, 25 mm NaHCO_3_, 3.0 mm KCl, 1.5 mm CaCl_2_, 1.0 mm NaH_2_PO_4_, and 1.0 mm MgCl_2._

### Extracellular recording of the field EPSP (fEPSP)

For electrophysiological recordings, slices were transferred to a recording chamber with recirculating aCSF (30.5 ± 1°C, equilibrated with 95% O_2_ and 5% CO_2_) and allowed 15 min to acclimate to the recording environment. The fEPSP in the CA1 was evoked by electrical stimulation. The stimulation electrode was positioned in Schaffer Collateral and the recording electrode (1–2 MΩ) was placed into the stratum radiatum of the CA1. The intensity of the electrical current (100–400 μ;A; 0.1–0.2 ms in duration) was set to the minimum amount of current required to generate ∼50% of the maximal initial slope (m_i_) of the fEPSP. The current stimulus used to examine the unpotentiated fEPSP was evoked at 700 μ;A (a stimulus intensity that evoked the maximal fEPSP amplitude in aCSF for all slices) and examined in aCSF, Mg^2+^-free aCSF, and Mg^2+^-free aCSF with 20 μm AP5 (Sigma-Aldrich). The composition of Mg^2+^-free aCSF: 119.5 mm NaCl, 10 mm glucose, 20 mm sucrose, 25 mm NaHCO_3_, 3.0 mm KCl, 1.5 mm CaCl_2_, and 1.0 mm NaH_2_PO_4_. The NaCl was increased to 119.5 mm to keep osmolarity from changing when switching from aCSF to Mg^2+^-free aCSF. The fEPSP was evoked every 20 s. After 10 min of recording the baseline fEPSP, long-term potentiation (LTP) was induced using high-frequency stimulation (HFS) or theta burst stimulation (TBS). HFS consisted four 500-ms trains of stimuli (100 Hz) given at 30-s intervals. TBS consisted of four trains of 10 bursts at 5 Hz, each burst was comprised of four pulses at 100 Hz. The fEPSP slope was normalized to baseline values (before HFS).

All recordings were made using the Multiclamp 700B (Molecular Devices: https://www.moleculardevices.com/systems/conventional-patch-clamp/multiclamp-700b-microelectrode-amplifier). Acquisition and *post hoc* analyses were performed using the Axon pCLAMP10 software suite (Molecular Devices: https://www.moleculardevices.com/system/axon-conventional-patch-clamp/pclamp-11-software-suite).

### Western blotting

Western blot assays were performed using entire hippocampal tissue homogenates from control and IH exposed mice. Hippocampal tissue from animals exposed to IH was harvested for Western blot analysis ∼12–16 h following the end of the IH_10_ protocol.

For quantitative analysis of HIF1a (R&D Systems catalog #AF1935, RRID:AB_355064) and proliferating cell nuclear antigen (PCNA; Bethyl catalog #A300-276A, RRID:AB_263393) content. Stepwise separation of cytoplasmic and nuclear protein extracts was prepared by NE-PER nuclear and cytoplasmic extraction kit (Thermo Scientific, 78833) by following manufacturer instructions. Briefly, cytoplasmic fragment was obtained by homogenizing tissue using a tissue grinder and then by pipetting in cytoplasmic extraction buffers. After isolation of cytoplasmic fragment, the insoluble pellet that contains nuclear proteins was suspended in nuclear extraction buffer and separated by centrifugation. Halt Protease Inhibitor (Thermo Scientific, 1860932) was added into cytoplasmic and nuclear extraction buffers to prevent protein degradation. Analyses for HIF1a and PCNA proteins were conducted by Raybiotech, using the automated capillary electrophoresis immunoassay machine (WES, ProteinSimple). The samples, blocking reagent, wash buffer, primary antibodies, secondary antibodies, and chemiluminescent substrate were dispensed into designated wells in the manufacturer provided microplate. After plate loading, the separation electrophoresis and immunodetection steps took place in the capillary system and were fully automated. Auto Western blot analysis was conducted at room temperature, and instrument default settings were used.

Quantitative Western blot analysis for GluN1, PSD-95, NADPH oxidase 4 (NOX4), and GAPDH were performed from hippocampal homogenates homogenized using either M-PER TM (Thermo Fisher Scientific) or RIPA buffer (Thermo Fisher Scientific) in the presence of protease and phosphatase inhibitors (Thermo Fisher Scientific) in cold ice. Samples were centrifuged at 14 rpm for 15 min at 4°C and the pellet was discarded. Samples were boiled for 15 min in loading buffer (Bio-Rad) at 60°C before loading 20- to 25-μ;g protein onto 4–20% Mini-PROTEAN TGX Stain-Free TM Protein Gels (Bio-Rad) and electrophoresed (120 V for 100 min) using Tris/glycine/SDS buffer (Bio-Rad). Gels were transferred to PVDF membrane (Bio-Rad) using Transfer-Blot Turbo System (Bio-Rad). Membranes were subsequently blocked (1 h, room temperature) with 5% non-fat milk (Bio-Rad) or 5% bovine serum albumin (BSA; Sigma-Aldrich) in Tris-buffered saline (Bio-Rad). Membranes were incubated (at 4°C overnight in 5% non-fat milk or BSA) under constant shaking with primary antibodies: monoclonal rabbit anti GluN1 (1:2000, Abcam), monoclonal rabbit anti-PSD-95 (1:1000, Cell signal), monoclonal rabbit anti- NOX4 (1:2000, Abcam), or monoclonal mouse anti-GAPDH (1:5000, Jackson ImmunoResearch). After washing three times with Tris-buffered saline-Tween 0.2% for 15 min, membranes were incubated (1 h, room temperature) with the appropriate secondary antibodies. Finally, membranes were washed three times with Tris-buffered saline-Tween 0.2% for 15 min, and immunoreactive proteins were detected with enhanced chemiluminescence (ECL) reagents according to manufacturer instructions (Bio-Rad). Signals were captured with the ChemiDoc system (Bio-Rad). The ImageJ image program (National Institutes of Health) was used to quantify optical band intensity.

### Protein carbonyls

Whole-cell protein lysates were isolated from hippocampal tissues by using M-PER mammalian protein extraction reagent (Thermo Scientific, 78501) and by adding Halt Protease Inhibitor (Thermo Scientific, 1860932). Protein lysates were immediately processed or kept in −80°C until used. The amount of protein carbonyls was determined using a Protein Carbonyl Colorimetric Assay kit (Cayman Chemical, catalog #10005020), per manufacturer instructions and absorbance was measured at a wavelength between 360–385 nm using a plate reader. Protein content was determined using a Protein Determination kit (Cayman Chemical, catalog #704002).

### Experimental design and statistical analyses

All n values are total number of animals, unless otherwise noted. Statistics were performed using Origin 8 Pro (OriginLab, RRID:SCR_014212) or Prism 6 (GraphPad Software; RRID:SCR_015807). Comparisons between two groups were conducted using unpaired two-tailed *t* tests with Welch’s correction. To compare three or more groups, a one-way ANOVA was performed followed by *post hoc* Dunnett’s test comparing experimental groups to control. Results are presented as single data points from each individual experiment and/or as the mean ± SEM. Significance was considered when *p* < 0.05. See Table 1 for statistical information related to analyses presented in this study.

**Table 1:** Description of statistical tests and associated values used throughout the study

Figure	Statistical test	Statistical values
1*A*	Unpaired *t* test with Welch's correction	*p* = 0.03; *t* = 2.789, df = 3
1*B*, left	One-way ANOVA, Dunnett's multiple comparison test	One-way ANOVA *p* = 0.0044, *F* = 7.191, 1 vs 2 (training session): *p* < 0.05, CI of diff = 23.74–217.9; 1 vs 3 (training session): *p* < 0.01, CI of diff = 47.11–241.3
1*B*, right	One-way ANOVA, Dunnett's multiple comparison test.	One-way ANOVA *p* = 0.0006, *F* = 11.68, 1 vs 2 (training session): *p* < 0.01, CI of diff = 63.81–239.3; 1 vs 3 (training session): *p* < 0.001, CI of diff = 66.88–242.3
1*C*, left	Unpaired *t* test with Welch's correction	*p* = 0.04, *t* = 2.85, df = 9
1*C*, right	Unpaired *t* test with Welch's correction	*p* = 0.03, *t* = 2.501, df = 9
1*D*	Unpaired *t* test with Welch's correction	*p* = 0.0037; *t* = 3.436, df = 15
1*E*	Unpaired *t* test with Welch's correction	*p* = 0.84; *t* = 0.2118, df = 5
1*F*, left	One-way ANOVA, Dunnett's multiple comparison test	One-way ANOVA *p* = 0.0136, *F* = 6.288, 1 vs 2 (training session): *p* > 0.05, CI of diff = –12.74 to 207.3; 1 vs 3 (training session): *p* < 0.01, CI of diff = 44.13–264.2
1*F*, right	One-way ANOVA, Dunnett's multiple comparison test	One-way ANOVA *p* = 0.0156, *F* = 5.688, 1 vs 2 (training session): *p* > 0.05, CI of diff = –3.202 to 221.3; 1 vs 3 (training session): *p* < 0.05, CI of diff = 36.56–261.1
1*G*, left	Unpaired *t* test with Welch's correction	*p* = 0.547, *t* = 0.6258, df = 9
1*G*, right	Unpaired *t* test with Welch's correction	*p* = 0.48, *t* = 0.7431, df = 9
1*H*, left	Unpaired *t* test with Welch's correction	*p* = 0.2120, *t* = 1.356, df = 8
2*A*	One-way ANOVA, Dunnett's multiple comparison test	One-way ANOVA *p* = 0.0004, *F* = 10.20; control vs AP5: *p* < 0.01, CI of diff = 21.28–62.13; control vs 10-IH: *p* < 0.01, CI of diff = 5.21–44.71; control vs 10-IH+AP5: *p* < 0.01, CI of diff = 11.04–51.89
2*B*	One-way ANOVA, Dunnett's multiple comparison test	One-way ANOVA *p* < 0.0001, *F* = 116.9; control vs AP5: *p* < 0.01, CI of diff = 54.80–80.72; control vs 10-IH: *p* < 0.01, CI of diff = 56.41–82.32
2*C*	Unpaired *t* test with Welch’s correction	*p* = 0.94; *t* = 0.065, df = 13.14
2*D*	One-way ANOVA, Dunnett's multiple comparison test	One-way ANOVA *p* < 0.0001, *F* = 54.50; 0-HIF1a+/− vs 10-HIF1a+/−: *p* > 0.05, CI of diff = –20.49 to 10.15; 10-HIF1a+/− vs 10-HIF1a+/−+ AP5: *p* < 0.01, CI of diff = 42.75–75.42
3*B*, top	One-way ANOVA, Dunnett's multiple comparison test	One-way ANOVA *p* = 0.56, *F* = 0.70; control vs IH_10_: *p* > 0.05, CI of diff = –24.53 to 54.60; control vs 0-HIF1a+/−: *p* > 0.05, CI of diff = –20.36 to 54.60; control vs 10-HIF1a+/−: *p* > 0.05, CI of diff = –33.12 to 39.06
3*B*, bottom	One-way ANOVA, Dunnett's multiple comparison test	One-way ANOVA *p* = 0.56, *F* = 0.70; control vs IH_10_: *p* < 0.05, CI of diff = –70.53 to –6.241; control vs 0-HIF1a+/−: *p* > 0.05, CI of diff = –56.75 to 3.840; control vs 10-HIF1a+/−: *p* > 0.05, CI of diff = –40.38 to 17.97
3*C*	One-way ANOVA, Dunnett's multiple comparison test	One-way ANOVA *p* = 0.014, *F* = 4.74; control vs IH_10_: *p* < 0.05, CI of diff = 0.96–0.78 ; control vs 0-HIF1a+/−: *p* > 0.05, CI of diff = –0.38 to 0.05; control vs 10-HIF1a+/−: *p* > 0.05, CI of diff = –0.27 to 0.157
3*D*	One-way ANOVA, Dunnett's multiple comparison test	One-way ANOVA *p* = 0.14, *F* = 2.39; control vs IH_10_: *p* > 0.05, CI of diff = –0.42 to 0.27 ; control vs 0-HIF1a+/−: *p* > 0.05, CI of diff = –0.63 to 0.0636; control vs 10-HIF1a+/−: *p* > 0.05, CI of diff = –0.35 to 0.33
4*A*	One-way ANOVA, Dunnett's multiple comparison test	One-way ANOVA *p* = 0.006 *F* = 6.871, control vs IH_10_: *p* < 0.01 CI of diff = –105.1 to –17.65; control vs 0-HIF1a+/−: *p* > 0.05 CI of diff = –52.12 to 35.32; control vs 10-HIF1a+/−: *p* > 0.05 CI of diff = –40.58 to 46.85
4*B*	One-way ANOVA, Dunnett's multiple comparison test	One-way ANOVA *p* = 0.003, *F* = 11.70; control vs IH_10_: *p* > 0.05, CI of diff = –1.85 to –0.28; control vs 0-HIF1a+/−: *p* > 0.05, CI of diff = –0.35 to 0.45; control vs 10-HIF1a+/−: *p* > 0.05, CI of diff = –0.28 to 0.52
5*A*	One-way ANOVA, Dunnett's multiple comparison test	One-way ANOVA *p* = 0.0023, *F* = 10.00; control vs IH_10_: *p* < 0.01, CI of diff = 0.09–0.75; control vs 10-MnTMPyP: *p* > 0.05, CI of diff = –0.49 to 0.26
5*B*	One-way ANOVA, Dunnett's multiple comparison test	One-way ANOVA *p* < 0.0001, *F* = 57.60, control vs IH: *p* < 0.001, CI of diff = 50.58–88.15; control vs IH+MnTMPyP: *p* > 0.05, CI of diff = –19.59 to 17.98
5*C*, top	One-way ANOVA, Dunnett’s multiple comparison test	One-way ANOVA *p* = 0.0008, *F* = 6.32, 1 vs 2 (training session): *p* > 0.05, CI of diff = –40.99 to 139.5; 1 vs 3 (training session): *p* < 0.001, CI of diff = 71.59–252.1
5*C*, bottom	One-way ANOVA, Dunnett’s multiple comparison test	One-way ANOVA *p* = 0.0056, *F* = 9.10, 1 vs 2 (training session): *p* > 0.05, CI of diff = –25.40 to 187.3; 1 vs 3 (training session): *p* < 0.01, CI of diff = 55.75–268.4
5*D*	Unpaired *t* test with Welch’s correction	*p* = 0.0005; *t* = 4.292, df = 16.7112

## Results

**Figure 1.**
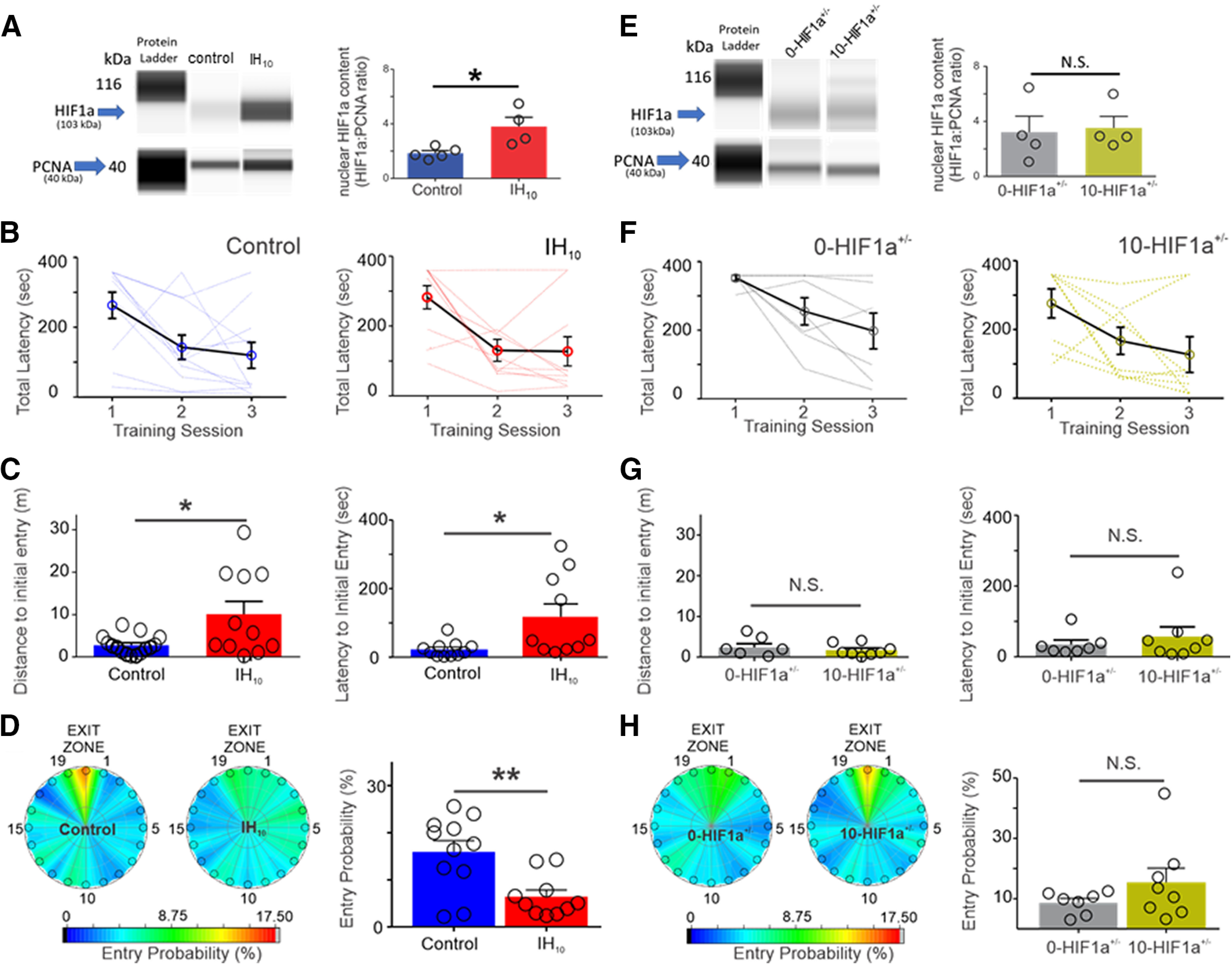
Ten days of IH increases hippocampal HIF1a and disrupts Barnes maze performance in wild-type mice but not in HIF1a+/−. ***A***, left, Representative digitized Western blotting images for HIF1a (103 kDa) and PCNA (40 kDa) in hippocampal nuclear protein fractions from control (*n* = 4) and IH_10_ (*n* = 4). Right, Quantification of HIF1a protein normalized to PCNA revealed that nuclear HIF1a was increased in IH_10_ when compared with control (*p* = 0.019). ***B***, Total latency to exit the Barnes maze during three training sessions in control (*n* = 10) and in IH_10_ (*n* = 11). Each blue (control) and red (IH_10_) line represents an individual performance during training. Training to the exit was conducted over three sessions. Each session was separated by 24 hours. ***C***, Left, During the probe trial, the distance traveled to initially enter the exit zone was shorter in control when compared with IH_10_ (*p* = 0.048). Right, Latency to initial entry was smaller in control as well (*p* = 0.034). ***D***, Heat maps of the mean entry probability across all false exits (1–19) and the exit zone during probe trial for the control and IH_10_. Comparison of entry probability into the exit zone during the probe trial reveals that control has a greater probability for entering the exit zone when compared with IH_10_ (*p* = 0.004). ***E***, Left, Representative digitized Western blotting images HIF1a and PCNA in hippocampal nuclear protein fractions from 0-HIF1a^+/−^ (*n* = 4) and 10-HIF1a^+/−^ (*n* = 4). Right, Quantification of HIF1a protein normalized to PCNA revealed that nuclear HIF1a is similar between 0-HIF1a^+/−^ and 10-HIF1a^+/−^ (*p* = 0.84). ***F***, Total latency to exit the Barnes maze during three training sessions in 0-HIF1a^+/−^ (*n* = 7) and in 10-HIF1a^+/−^ (*n* = 8). Each gray (0-HIF1a^+/−^) and yellow (10-HIF1a^+/−^) line represents an individual performance during training. All experimental groups exhibit decreased total latency over the course of training. ***G***, Left, In HIF1a^+/−^, the distance initial to initial entry into the exit zone was similar between 0-HIF1a^+/−^ and 10-HIF1a^+/−^ (*p* = 0.55). Right, Latency to initial entry into the exit zone during the probe trial were similar between 0-HIF1a^+/−^ and 10-HIF1a^+/−^ (*p* = 0.39). ***H***, Heat maps of the mean entry probability into all zones during the probe trial for 0-HIF1a^+/−^ and 10-HIF1a^+/−^. Entry probability was similar between 0-HIF1a^+/−^ and 10-HIF1a^+/−^ (*p* = 0.21); *p < 0.05; **p < 0.01; N.S., *p* > 0.05.

HIF1a protein content was measured in nuclear extracts prepared from wild-type mice unexposed to IH (control) and exposed to 10 d of IH (IH_10_). Nuclear HIF1a was approximately two times greater in extracts from IH_10_ than control (control *n* = 4, IH_10_ *n* = 4; Fig. 1*A*). To determine the behavioral consequences of IH, we examined spatial learning and memory by assessing performance in a Barnes maze in control (*n* = 11) and IH_10_ (*n* = 10). During training, control and IH_10_ exhibited progressive improvement on locating the exit zone as indicated by the decrease in latency to exit over course of three training sessions and was similar between groups (Fig. 1*B*).

In the probe trial (when the exit was closed), no difference was evident between the total distance traveled between control and IH_10_ (control: 25.22 ± 1.74 m vs IH_10_: 27.91 ± 2.21 m, *p* = 0.35; data not shown) suggesting no locomotor differences between groups. However, performance in locating the exit zone was different between control and IH_10_ as the distance to initial entry to the exit zone was greater in IH_10_ (control: 2.60 ± 0.70 m vs IH_10_: 10.34 ± 3.32 m, *p* = 0.048; Fig. 1*C*), and a larger latency to initial entry exit zone was observed in IH_10_ (control: 22.60 ± 6.28 s vs IH_10_: 117.90 ± 37.47 s, *p* = 0.034; Fig. 1*C*). Additionally, when comparing the probability to exit zone entry, the control group consistently discriminated the location of exit hole against the other holes, yet this was not apparent in IH_10_ (control: 15.93 ± 2.39% vs IH_10_: 6.44 ± 1.38%, *p* = 0.0037; Fig. 1*D*). Together, these findings indicated that wild-type animals exposed to IH have increased expression of HIF1a and impairments to spatial memory.

Nuclear HIF1a protein content was similar between extracts from hippocampi of HIF1a^+/−^ mice unexposed to IH (0-HIF1a^+/−^) when compared with HIF1a^+/−^ mice exposed to 10 d of IH (10-HIF1a^+/−^; 0-HIF1a^+/−^, *n* = 4, 10-HIF1a^+/−^ *n* = 4; Fig. 1*E*). In 0-HIF1a^+/−^ (*n* = 7), and 10-HIF1a^+/−^ (*n* = 8) performance in the Barnes maze was similar over the course of the training sessions (Fig. 1*F*). During the probe trial, the total distance traveled by 0-HIF1a^+/−^ to 10-HIF1a^+/−^ were similar (0-HIF1a^+/−^ = 19.47 ± 1.61 m, 10-HIF1a^+/−^ = 22.42 ± 1.61 m; *p* = 0.55; data not shown) suggesting no locomotor differences between groups. Moreover, the distance to initial entry to the exit zone (0-HIF1a^+/−^ = 2.37 ± 0.91 m, 10-HIF1a^+/−^ = 1.71 ± 0.50 m; *p* = 0.54; Fig. 1*G*), latency to initial entry into the exit zone (0-HIF1a^+/−^ = 35.18 ± 12.28 s, 10-HIF1a^+/−^ = 57.28 ± 27.08 s; *p* = 0.48; Fig. 1*G*); and the entry probability into the exit zone (0-HIF1a^+/−^ = 8.75 ± 1.38%, 10-HIF1a^+/−^ = 15.51 ± 4.73%; *p* = 0.21; Fig. 1*H*) for 0-HIF1a^+/−^ and 10-HIF1a^+/−^ were similar between both groups. These data demonstrate that in HIF1a^+/−^ mice the IH-dependent increase in nuclear HIF1a protein was mitigated, and spatial memory was unaffected by IH. Furthermore, these data raise the possibility that increased hippocampal nuclear HIF1a signaling causes deficits to hippocampal LTP.

**Figure 2.**
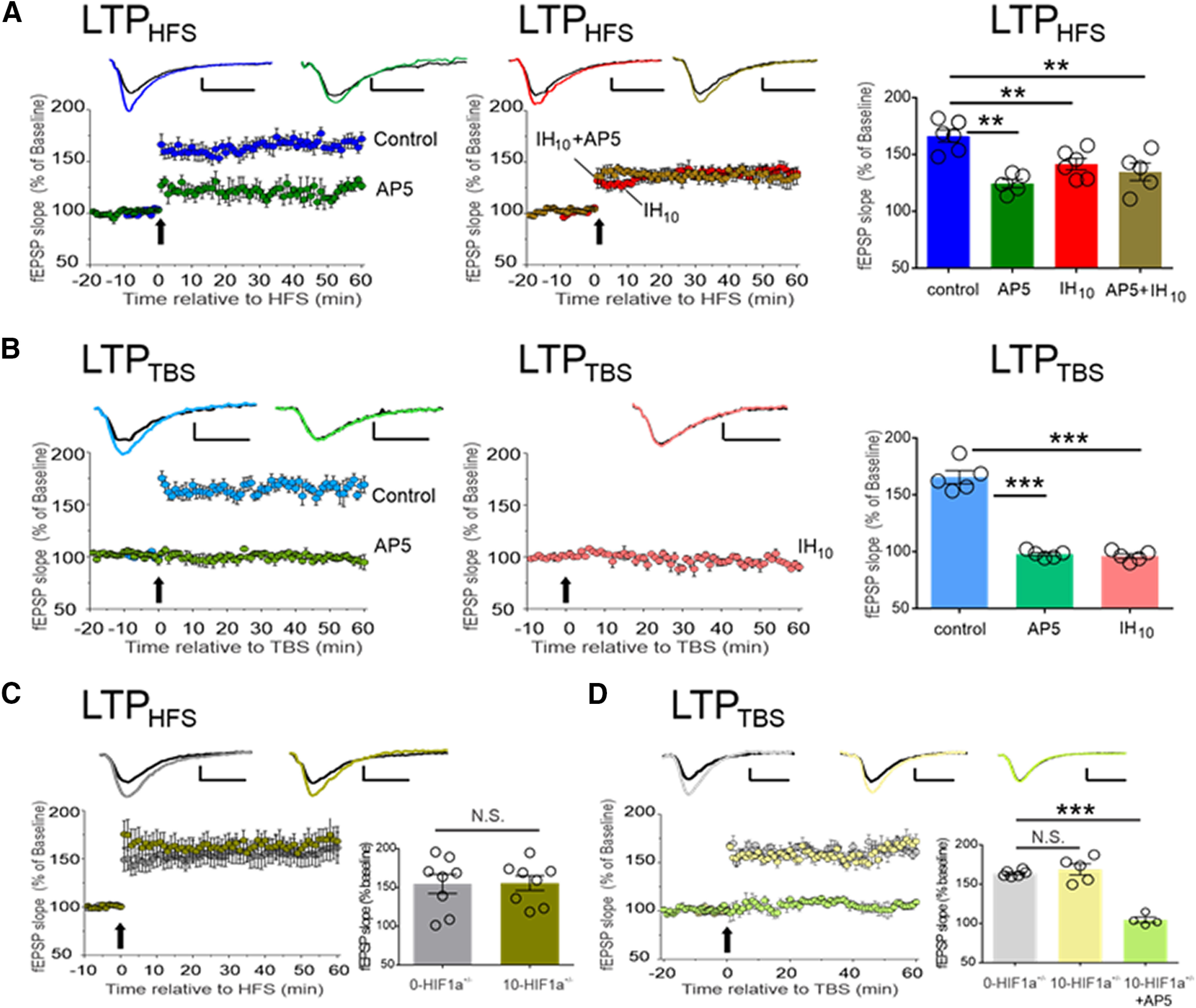
IH suppresses NMDAr-dependent synaptic potentiation in wild-type hippocampal slices, but NMDAr-dependent LTP is unaffected by IH in the hippocampal slices from HIF1a^+/−^. ***A***, LTP was evoked using HFS in control (blue, *n* = 6) is attenuated by AP5 (green, *n* = 5). LTP_HFS_ is attenuated in following IH (IH_10_, red, *n* = 6) and is no longer sensitive to AP5 (IH+AP5, gold, *n* = 5). A comparison of LTP_HFS_ magnitude (60 min following HFS) was performed to compare experimental conditions to control; **p < 0.01. ***B***, LTP_TBS_ is readily evoked in control (light blue, *n* = 5) and is completely blocked by AP5 (light green, *n* = 5). Following IH, LTP_TBS_ is present (IH_10_, pink, *n* = 5). Following a one-way ANOVA, a *post hoc* comparison of LTP_TBS_ magnitude (60 min following TBS) was performed to compare experimental conditions to control; ***p < 0.01. ***C***, LTP_HFS_ was evoked in both 0-HIF1a^+/−^ (*n* = 8, gray) and 10-HIF1a^+/−^ (*n* = 8, dark yellow). No difference was found when comparing LTP_HFS_ magnitude between 0-HIF1a^+/−^ and 10-HIF1a^+/−^ (*p* = 0.94). ***D***, LTP_TBS_ was evoked in both 0-HIF1a^+/−^ (*n* = 6, light gray), 10-HIF1a^+/−^ (*n* = 5, light yellow), and 10-HIF1a^+/−^ + AP5 (*n* = 5, light green). No difference was found when comparing LTP_TBS_ magnitude of 0-HIF1a^+/−^ and 10-HIF1a^+/−^. Representative traces illustrate baseline (black) and 60 min following HFS (colored trace). Scale bars: 0.2 mV/10 ms. In experiments using AP5, electrophysiological recordings began at 20 min before eliciting LTP (i.e., *t* = −20) while AP5 was applied 10 before eliciting LTP (i.e., *t* = −10). For all the experiment, the arrow represents the electric protocols: HFS or TBS; ***p < 0.001, **p < 0.01; N.S., *p* > 0.05.

The mechanisms underlying LTP are key substrates for learning and memory. We, therefore, examined LTP from area CA1 in hippocampal in brain slices from control and IH_10_. LTP from control was consistently evoked by HFS (LTP_HFS_; Fig. 2*A*, blue, *n* = 6). NMDAr blockade with AP5 attenuated LTP_HFS_ magnitude but did not prevent the occurrence of the phenomenon (Fig. 2*A*, green, *n* = 5). These findings demonstrated that both NMDAr-dependent and NMDAr-independent mechanisms contributed to the generation of LTP_HFS_. Following IH, LTP_HFS_ was smaller in magnitude (Fig. 2*A*, red, *n* = 6) and was no longer sensitive to AP5 (Fig. 2*A*, gold, *n* = 5).

We next examined whether IH prevented another LTP evoked by TBS (LTP_TBS_; Fig. 2*B*, light blue, *n* = 5), a form of synaptic potentiation dependent on the NMDAr, as AP5 prevent LTP_TBS_ (Fig. 2*B*, light green, *n* = 5). Following IH, LTP_TBS_ could no longer be evoked (Fig. 2*B*, pink, *n* = 5).

In contrast to the wild type, LTP_HFS_ was similar in 0-HIF1a^+/−^ (Fig. 2*C*, gray *n* = 8) and in 10-HIF1a^+/−^ (Fig. 2*C*, dark yellow, *n* = 8). In the hippocampal brain slice, the magnitude of LTP_TBS_ was similar between 0-HIF1a^+/−^ (Fig. 2*D*, light gray, *n* = 6) and in 10-HIF1a^+/−^ (Fig. 2*D*, light yellow, *n* = 5). Additionally, AP5 blocked LTP_TBS_ in the 10-HIF1a^+/−^ (Fig. 2*D*, light green, *n* = 4). Together, these findings suggested that IH-dependent HIF1a signaling suppresses NMDAr-dependent potentiation by disrupting the NMDAr physiology. To test this, we examined the contribution of the NMDAr to the unpotentiated fEPSP.

**Figure 3.**
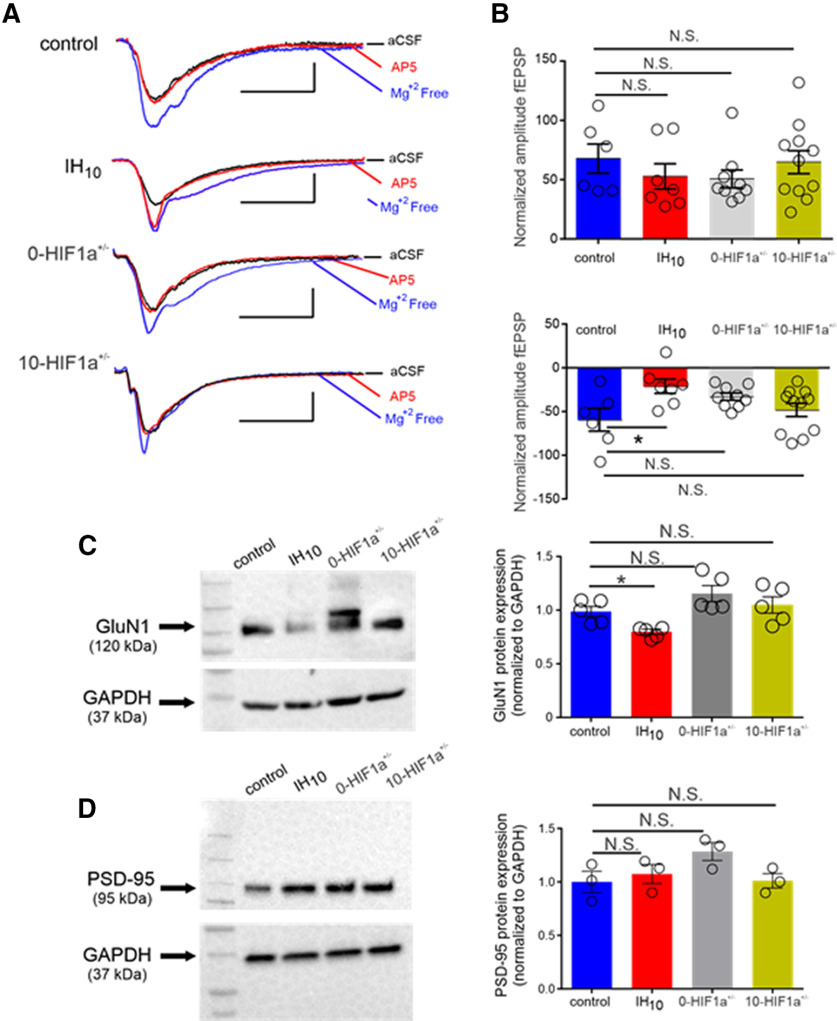
The IH reduces the contribution of the NMDAr to fEPSP and GluN1 protein from wild-type mice but does not induce these changes in HIF1a^+/−^. ***A***, Representative traces of the fEPSP from control, IH_10_, 0-HIF1a^+/−^, and 10-HIF1a^+/−^ in: aCSF (black), Mg^2+^-free media (blue), and Mg^2+^-free media with AP5 (red). Scale bars: 0.4 mV/10 ms. ***B***, top, Change in amplitude of the fEPSP from aCSF to Mg^2+^-free media. Bottom, Change in amplitude of the fEPSP from Mg^2+^-free media to Mg^2+^-free media with AP5; *p < 0.05; N.S., *p* > 0.05. ***C***, left, Representative Western blottings of GluN1 and the housekeeping protein, GAPDH from control (*n* = 5), IH_10_ (*n* = 5), 0-HIF1a^+/−^ (*n* = 5), and 10-HIF1a^+/−^ (*n* = 5). Right, Comparisons of normalized GluN1 protein expression were performed to compare experimental conditions to control. This revealed that GluN1 was reduced in IH_10_ and unchanged in both 0-HIF1a^+/−^ and 10-HIF1a^+/−^; *p < 0.05; N.S., *p* > 0.05. ***D***, left, Representative Western blottings of PSD-95 and the housekeeping protein, GAPDH from control (*n* = 3), IH_10_ (*n* = 3), 0-HIF1a^+/−^ (*n* = 3), and 10-HIF1a^+/−^ (*n* = 3). Right, Comparisons of normalized PSD-95 protein expression were performed to compare experimental conditions to control; *p < 0.05; N.S., *p* > 0.05.

A fEPSP with maximal amplitude in aCSF (fEPSP_max_) was evoked using saturating current stimulus (700 μA; Fig. 3*A*, black, aCSF) in control (*n* = 6, fEPSP_max_= −1.05 ± 0.14 mV), IH_10_ (*n* = 7, fEPSP_max_= −0.85 ± 0.08 mV), 0-HIF1a^+/−^ (*n* = 9, fEPSP_max_= −0.941 ± 0.04 mV), and 10-HIF1a^+/−^ (*n* = 11, fEPSP_max_= −0.90 ± 0.06 mV). When compared with control, no difference in fEPSP_max_ was observed from any experimental group (one-way ANOVA: *p* = 0.39, *F* = 1.035; control vs IH_10_: *p* > 0.05, 95% CI of diff = −0.4960 to 0.09,542; control vs 0-HIF1a^+/−^: *p* > 0.05, 95% CI of diff = −0.3938 to 0.1665; 10-HIF1a^+/−^: *p* > 0.05, 95% CI of diff = −0.4219 to 0.1176; data not shown). Switching to Mg^2+^-free aCSF relieved the Mg^2+^ blockade of existing NMDAr and caused the fEPSP to increase all groups (Fig. 3*A*, blue, Mg^2+^ free). The change in fEPSP amplitude from aCSF to Mg^2+^ free was not different when comparing the other experimental groups to control (Fig. 3*B*, top). However, the NMDAr antagonist, AP5, reduced the fEPSP in 0-HIF1a^+/−^ and 10-HIF1a^+/−^ similar to that of control (Fig. 3*A*, red, AP5) yet was less effective in IH_10_ (Fig. 3*B*, bottom). These findings suggested that IH suppressed contribution of the conductance of NMDAr within neurons in a HIF1a dependent manner. Such an effect could be due to direct effects on unitary conductance of the NMDAr or by the down regulation of the receptor itself. Therefore, we examined whether expression of the GluN1, the obligatory subunit of the NMDAr, was disrupted by IH.

We compared GluN1 subunit expression in control (*n* = 5), IH_10_ (*n* = 5), 0-HIF1a^+/−^ (*n* = 5), and 10-HIF1a^+/−^ (*n* = 5). IH reduced GluN1 in wild-type hippocampi, yet GluN1 expression was similar to control in both 0-HIF1a^+/−^ or 10-HIF1a^+/−^ (Fig. 3*C*). This reduction in GluN1 may have resulted from an IH-mediated reduction in synapse. Therefore, we sought to determine whether IH caused a reduction in a scaffolding protein of the glutamatergic synapse PSD-95 (Fig. 3*D*, *n* = 3 per group). When compared with control, no difference in PSD-95 was detected any experimental group (Fig. 3*D*). These findings together indicated that IH-dependent HIF1a signaling specifically likely targets a reduction of the NMDAr by suppressing GluN1 expression without causing gross reductions in glutamatergic synapses. Such a reduction in NMDAr expression would likely contribute to the reduced sensitivity to AP5 following IH and contribute to impaired NMDAr-dependent LTP.

**Figure 4.**
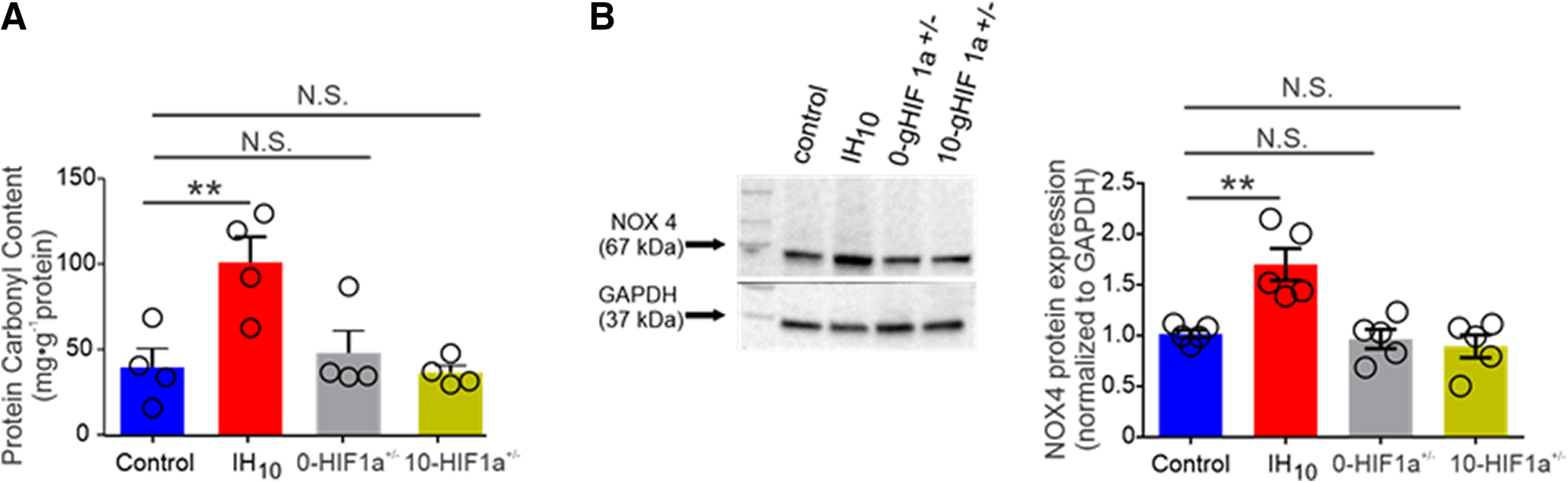
IH enhances protein carbonyl content and increase NOX4 expression in wild type but not in HIF1a^+/−^. ***A***, Hippocampal homogenates from control (*n* = 4), IH_10_ (*n* = 4), 0-HIF1a^+/−^ (*n* = 4), and 10-HIF1a^+/−^ (*n* = 4). While IH_10_ displayed elevated protein, carbonyl content was not elevated in either 0-HIF1a^+/−^ or 10-HIF1a^+/−^. ***B***, Comparison of the pro-oxidant enzyme, NOX4, from control (*n* = 5), IH_10_ (*n* = 5), 0-HIF1a^+/−^ (*n* = 5), and 10-HIF1a^+/−^ (*n* = 5) reveals that NOX4 is increased in IH_10_; *p* < 0.01), but not elevated in either 0-HIF1a^+/−^ or 10-HIF1a^+/−^; **p < 0.01; N.S., *p* > 0.05.

As IH-dependent HIF1a signaling can lead to a pro-oxidant condition, we next sought to determine whether IH-dependent HIF1a signaling enhanced ROS production within the hippocampus. Protein carbonyl content in hippocampal homogenates from control (*n* = 4), IH_10_ (*n* = 4), 0-HIF1a^+/−^ (*n* = 4), and 10-HIF1a^+/−^ (*n* = 4) revealed that protein carbonyl content was elevated in IH_10_ yet unchanged changed in homogenates from either 0-HIF1a^+/−^ or 10-HIF1a^+/−^ (Fig. 4*A*). NOX4 is a ROS generating protein that can be transcriptionally regulated by HIF1a (Diebold et al., 2010). Therefore, we next determined Nox4 expression in hippocampal homogenates from control (*n* = 5), IH_10_ (*n* = 5), 0-HIF1a^+/−^ (*n* = 5), and 10-HIF1a^+/−^ (*n* = 5). NOX4 was elevated in IH_10_ yet unchanged changed in homogenates from either 0-HIF1a^+/−^ or 10-HIF1a^+/−^ (Fig. 4*B*). Together, these data suggest that enhanced ROS production by IH-dependent HIF1a signaling involves the upregulation of NOX4. However, IH-dependent ROS production was involved with the changed expression of GluN1 remained uncertain.

**Figure 5.**
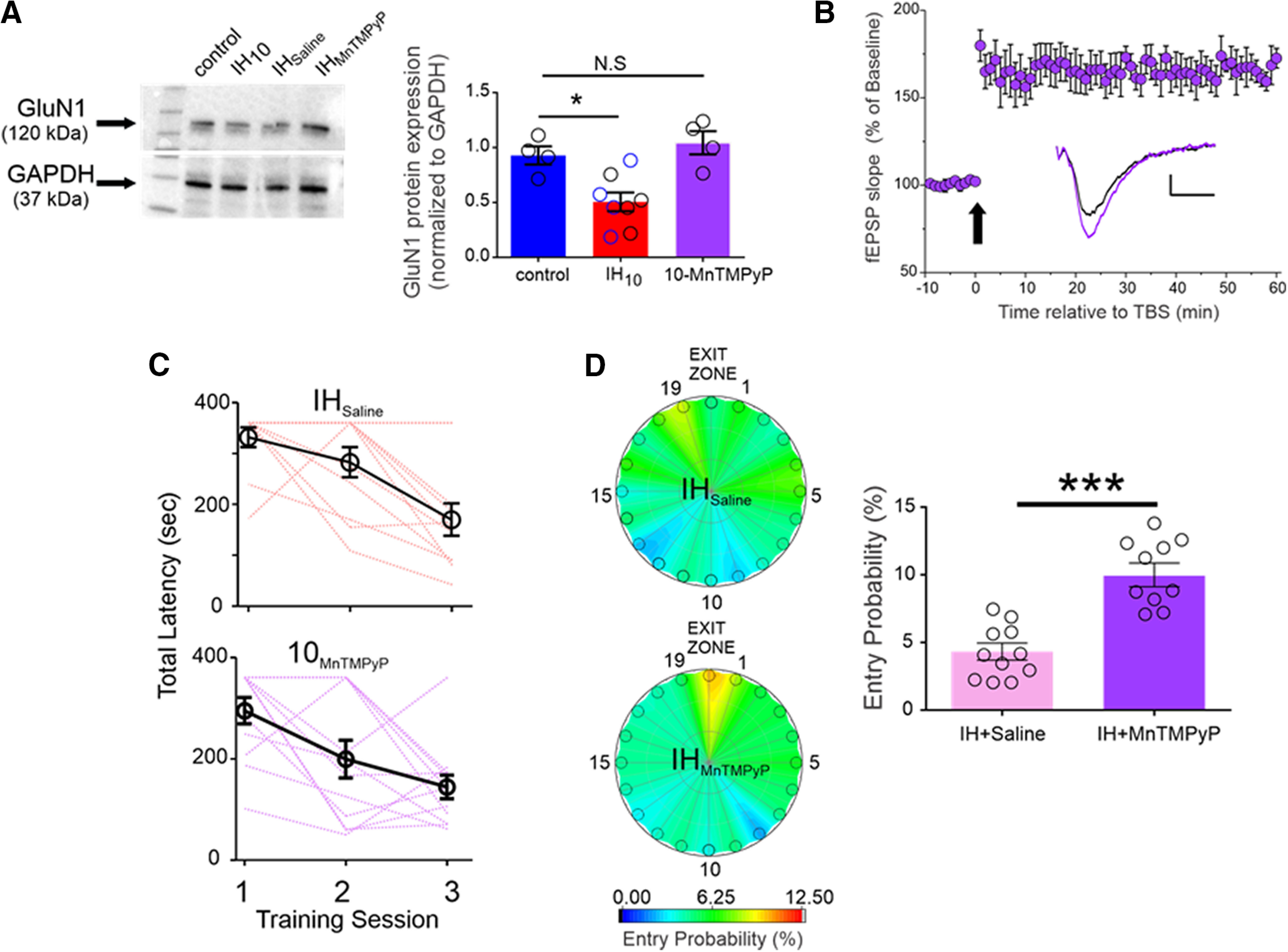
Antioxidant treatment mitigates the IH-dependent effects on GluN1 expression, LTP_TBS,_ and performance in the Barnes maze. ***A***, left, Representative Western blottings of GluN1 and GAPDH from Control, IH_10_, wild-type mice treated with saline during 10 d of IH (i.e., vehicle control exposed to IH, IH_Saline_, *n* = 4), wild-type mice treated with MnTMPyP during 10 d of IH (IH_MnTMPyP_). Right, Normalized GluN1 protein expression was examined in control (*n* = 4), IH_10_ (*n* = 4), IH_Saline_ (*n* = 4), IH_MnTMPyP_ (*n* = 4). No difference in GluN1 was evident between IH_10_ (open black circles in IH_10_ label) and IH_Saline_ (open blue circles in IH_10_ label); therefore, the two groups were merged into the IH_10_ label for comparisons to control. Comparisons revealed that GluN1 was reduced only in IH_10_ and unchanged in IH_MnTMPyP_. ***B***, In hippocampal slices from IH_MnTMPyP_, LTP_TBS_ (*n* = 5) could be reliably evoked contrasting the effect of IH_10_ on LTP_TBS_ (Fig. 2*B*). Scale bars: 0.2 mV/10 ms. The arrow represents the TBS protocol. ***C***, The total latency to exit the Barnes maze progressively decreased in both IH_Saline_ (*n* = 11, pink lines represent individual performance) and IH_MnTMPyP_ (*n* = 10, purple lines represent individual performance), suggesting that both groups could learn the exit zone location. ***D***, Heat maps of the mean entry probability across all false exits (1–19) and the exit zone during the probe trial for IH_Saline_ and IH_MnTMPyP_. Comparison of entry probability into the exit zone during the probe trial reveals that IH_MnTMPyP_ has a greater probability for entering the exit zone when compared with IH_Saline_ (*p* = 0.006); ***p < 0.001, *p < 0.05; N.S., *p* > 0.05.

To resolve the involvement of IH-dependent ROS production on the regulation of GluN1, protein homogenates were prepared from four groups: control (*n* = 4); IH_10_ (*n* = 4); wild-type mice administered saline during 10 d of IH exposure (IH_Saline_, *n* = 4); wild-type mice administered the superoxide anion scavenger, MnTMPyP, during IH (IH_MnTMPyP_, *n* = 4). GluN1 was reduced in IH_10_ and IH_Saline_; however, GluN1 from 10-MnTMPyP was similar to that of control (Fig. 5*A*), which coincided with the ability to evoke LTP_TBS_ from IH_MnTMPyP_ (*n* = 5; Fig. 5*B*). Behavioral performance was also assessed in IH_Saline_ (*n* = 11) and IH_MnTMPyP_ (*n* = 10). Both IH_Saline_ and IH_MnTMPyP_ exhibited a progressive improvement in locating the exit as indicated by the total latency to exit over the course of training (Fig. 5*C*). During the probe trail, the two groups exhibited similar values for distance to initial entry into the exit zone (IH_Saline_ = 0.29 ± 0.06 m, IH_MnTMPyP_ = 0.28 ± 0.05 m; *p* = 0.87; data not shown), and similar latency to initial entry into the exit zone (IH_Saline_ = 77.74 ± 24.42 s, IH_MnTMPyP_ = 26.00 ± 5.67 s; *p* = 0.06; data not shown), although the variance between the values for latency to initial entry was different between IH_Saline_ and IH_MnTMPyP_ (*F* = 17.00, DFn = 10, Dfd = 11; *p* < 0.0001; data not shown). Moreover, entry probability into the exit zone during the probe trial was greater in IH_MnTMPyP_ (IH_Saline_ = 4.33 ± 0.63%, IH_MnTMPyP_ = 10.32 ± 1.26%; *p* = 0.0005; Fig. 5*D*). These data indicated that scavenging IH-derived superoxide anion prevented the reduction in the obligatory subunit of the NMDAr, prevented the loss of LTP_TBS_, and mitigated behavioral deficits caused by IH.

## Discussion

Our study establishes a role for IH-dependent HIF1a signaling in impairing hippocampal neurophysiology that supports spatial memory. Consistent with previous reports indicating that IH impacts spatial memory (Row et al., 2002; Gozal et al., 2003), we observed that IH disrupted performance in the Barnes maze. The cognitive disruptions we observed coincided with enhanced nuclear HIF1a in the hippocampus, a shift toward a pro-oxidant state, and impairment to NMDAr-dependent LTP. We found that either heterozygosity in HIF1a and antioxidant administration prevented the effects of IH on the hippocampus. Together, these findings reveal a mechanistic pathway by which IH such as that experienced with sleep apnea impairs mechanisms underpinning spatial memory.

Evaluating the behavioral performance in control and IH_10_ showed that both groups progressively improved with training, yet prominent differences were present during the probe trial. These results suggested IH produced modest impairments to cognitive performance and is reminiscent of mild cognitive deficits documented among individuals suffering from sleep apnea (Wallace and Bucks, 2013; Devita et al., 2017b; Leng et al., 2017). These behavioral impairments coincided with targeted loss in NMDAr-dependent LTP after IH. However, neither the behavioral deficits nor impaired synaptic potentiation was observed in HIF1a^+/−^ exposed to IH implicating a role for IH-dependent HIF1a signaling in these phenomena.

Although administration of the prolyl hydroxylase inhibitor, dimethyloxalylglycine (DMOG), enhances HIF1a and coincides with the suppression of hippocampal LTP (Wall et al., 2014), this pharmacological approach for enhancing HIF1a can also disrupt cellular respiration well before the activation of HIF1a-dependent pathways (Zhdanov et al., 2015). This confounds understanding how enhanced HIF1a may impact hippocampal synaptic plasticity. Our experiments using HIF1a^+/−^ mice resolved this issue. Heterozygosity in HIF1a prevented the IH-dependent increase in NOX4, the ROS-producing enzyme transcriptionally regulated by HIF1a (Diebold et al., 2010). Increasing NOX4 would be expected to increase the production of ROS and, if left unchecked, promote a pro-oxidant state. Indeed, IH led to increased protein carbonylation, an indication of a shift toward a more pro-oxidant state. The HIF1a-dependent increase in pro-oxidant condition was presumably due to ROS production from the enhanced presence of NOX4. The pro-oxidant state suppressed NMDAr-dependent LTP and disrupted performance in the Barnes maze.

In agreement with a previous report (Gozal et al., 2001), our protein analyses indicated that IH reduced GluN1 expression, the obligatory subunit of the NMDAr. Alone, this observation could not discriminate whether the effect of IH on GluN1 expression reflected a reduction of the NMDAr at the glutamatergic synapse, a decline in extrasynaptic receptors, a premature degradation of GluN1 before assembly of the receptor or some combination of the three. The reduction in GluN1 was not accompanied by a reduction in PSD-95, suggesting that IH did not indiscriminately cause a loss of glutamatergic synapses. Following IH, the unpotentiated fEPSP (in Mg^2+^-free aCSF) was less sensitive to NMDAr blockade. Together, these findings may be interpreted as indicating that IH remodels the glutamatergic synapse by reducing receptor expression. Such a reduction in the synaptic NMDAr would likely disrupt NMDAr-dependent LTP. However, this may not be the only avenue by which IH disturbs NMDAr-based physiology.

Administration of MnTMPyP during IH prevented both GluN1 reduction and impairment to NMDAr-dependent LTP. Similarly, in 10-HIF1a^+/−^, GluN1 expression and NMDAr-dependent LTP was similar to that of control. These findings together indicate that HIF1a mediated ROS production is a principal mechanism that diminishes NMDAr function. While our experiments support the possibility that IH causes reduced receptor expression, the conductance of the NMDAr is known to be redox sensitive (Bodhinathan et al., 2010; Kumar and Foster, 2013). Specifically, oxidation of the NMDAr attenuates NMDAr conductance (Choi and Lipton, 2000; Lipton et al., 2002; Guidi et al., 2015; Foster et al., 2017). It is, therefore, likely that some combination of oxidative modulation and downregulation of the NMDAr mediates the disrupted NMDAr physiology caused by IH. However, we did not acutely manipulate redox state and do not know to what extent the two processes contribute IH-dependent effects on NMDAr activity. This remains an open question to be investigated.

Independent of the precise cause, changed NMDAr activity by IH likely decreases the NMDAr-dependent rise in intracellular Ca^2+^. While a rise intracellular Ca^2+^ is an important event for downstream intracellular signaling critical to LTP, it also is likely to mediate other Ca^2+^-dependent processes within the neuron. With respect to IH, ROS production can increase intracellular Ca^2+^ via the inositol 1,4,5-trisphosphate receptor (IP3R; Yuan et al., 2008), which then serves as a positive feedback mechanism to enhance and stabilize HIF1a signaling (Prabhakar and Semenza, 2012). As we observed that IH increases NOX4 and protein carbonyls in a HIF1a dependent fashion, excess elevations in intracellular Ca^2+^ within hippocampal cells may promote a feedforward mechanism that enhances HIF1a activity and ROS generation. Thus, the reduction of NMDAr activity may serve as a necessary phenomenon to minimize intracellular Ca^2+^ and prevent potential exacerbation of cellular stress if left unregulated.

The forced shift from NMDAr-dependent to NMDAr-independent forms of synaptic plasticity observed with IH is a phenomenon also found in models of the aging (Boric et al., 2008; Robillard et al., 2011). Thus, IH, like normal aging, limits the mechanisms normally used to support learning and memory in younger animals. Our work indicates that the HIF1a dependent pro-oxidant condition causes this aging phenotype. As the current study used younger animals (P50–P80), examining how IH affects mechanisms of learning and memory in aged subjects will be important to resolve.

**Figure 6.**
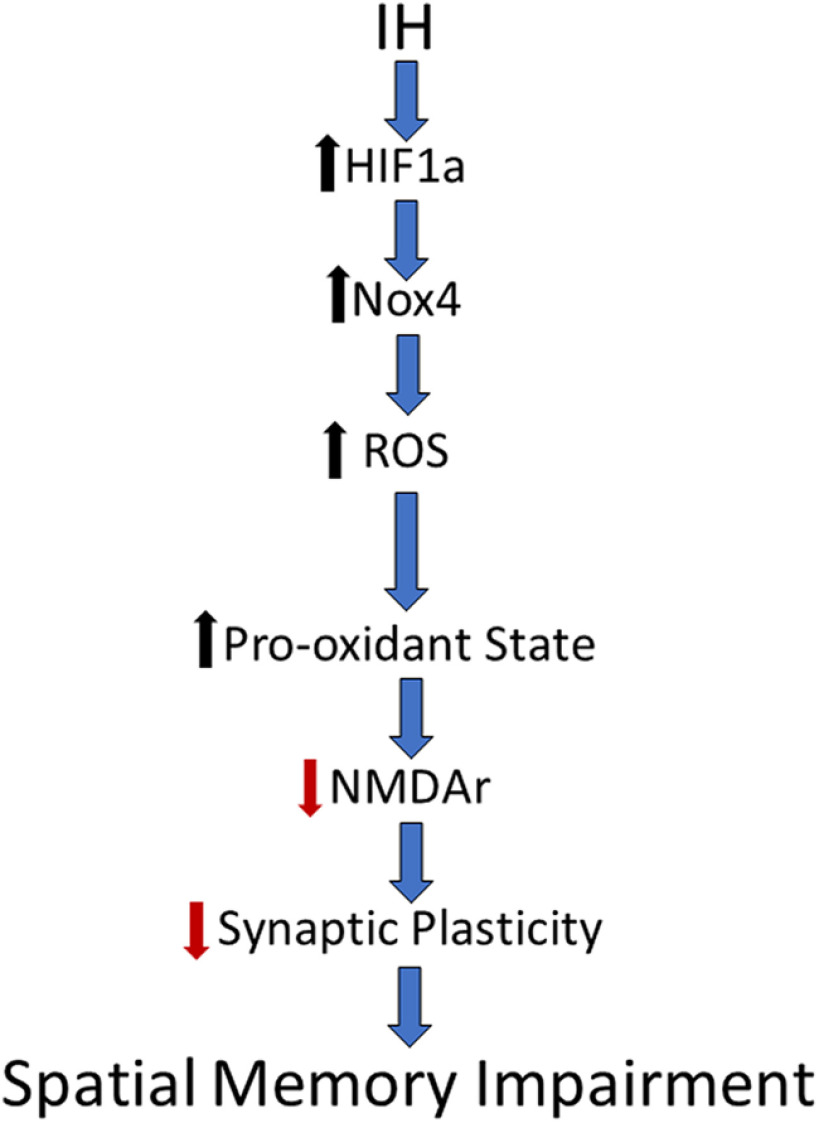
A mechanistic framework by which sleep apnea lowers the threshold for cognitive impairment. Schematic synthesizing our findings into a pathway by which IH promotes a pro-oxidant state in the hippocampus that impairs NMDAr-dependent plasticity and spatial memory.

In conclusion, we have identified an important pathway by which IH-dependent HIF1a signaling causes a pro-oxidant state that destabilizes hippocampal synaptic plasticity and disrupts spatial memory. We propose that these observations establish a mechanistic framework by which sleep apnea may lower of the threshold for cognitive impairment (Fig. 6). This mechanism may contribute to the emergence of neurologic diseases associated with untreated sleep apnea.

## Synthesis

Reviewing Editor: Bradley Postle, University of Wisconsin Decisions are customarily a result of the Reviewing Editor and the peer reviewers coming together and discussing their recommendations until a consensus is reached. When revisions are invited, a fact-based synthesis statement explaining their decision and outlining what is needed to prepare a revision will be listed below. The following reviewer(s) agreed to reveal their identity: Thomas Foster, Ashok Kumar.

Here is the full text of the reviews:

R1:

The authors did a nice job and have a fairly complete story. Below are suggestions to improve the manuscript. Major concerns relate to needed comparisons between WT and Hif1a+/- mice. In addition, I have concerns about the proposed link between impaired LTP, NMDAr function, and NMDAr expression.

Methods

For measures of nuclear Hif1a, when was the tissue harvested relative to treatment?

Barnes Maze began on the seventh day of IH10 exposure with respective controls run at the same time. Was the testing performed immediately after the treatment on day 7?

Define primary latency to exit zone versus initial latency.

Results

Is there a behavioral difference in WT and Hif1a+/-? It appears there may be a difference in learning for the controls (Fig 1c vs Fig1f).

One assumption is that the LTP in Hif1a+/- mice is due to NMDAr; however, this was not shown (i.e. include AP5). This data needs to be included.

It is difficult to measure decay time of NMDAr synaptic responses in extracellular recording and there are several problems in the current study.

1) One problem is the peak amplitudes are vastly different before and after application of AP5 in control mice. Was there a difference in the input/output relationship (AMPAr or NMDAr) between control and Hif1a+/- mice? Was there a difference in input/output curves before and after AP5? At the very least, was there a difference in the maximally evoked fEPSP across mouse strains or due to AP5 application? It appears that AP5 results in a large decrease in the amplitude for the WT control slices (Fig3A). This is not observed for IH10 or Hif1a+/- mice, which suggests fewer NMDARs are at the synapse following IH in WT mice and that synaptic NMDAr levels are low in Hif1a+/- mice regardless of treatment. The question of amplitude differences is also important for interpretation of western blot studies of subunit expression and possible synaptic loss (see Discussion comments).

2) Second, in order to measure the true decay, the responses should be normalized to the peak (i.e. peak = 100%). Thus, the peaks in each panel should lineup on the horizontal plane and decay differences would then be evident. In figure 3, the decay is contaminated by differences in the peak.

Discussion

Missing from the discussion is the mechanism for the decrease in LTP and apparent decrease in the NMADr response. The authors state: our findings indicate that IH reduces receptor expression causing targeted loss of NMDAr-dependent LTP through a HIF1a mediated pro-oxidant mechanism. Implicit in this statement is the assumption that the decrease in LTP is due to a decrease in NMDAr at the synapse.

First, the authors should discuss whether the reduction in NMDAr expression represents synapse loss, a decreased level of NMDAr at the synapse in the absence of synaptic loss, or loss from intracellular or extrasynaptic compartments. In this case, input/output curves of the fEPSP would help. If the AMPAr component of transmission is not altered, this would suggest no loss of synapses, but does not rule out redox state changes in NMDAr function. If the input/output curve was also decreased, it is possible that there is synaptic loss. In addition, it would have been helpful if the authors had a measure of synaptic number (e.g. PSD95 western blots).

Second, the authors did not test for a decrease in NMDAr function due to redox state as reported by the Lipton and Foster groups. In this case, the authors would have needed to determine the effects of oxidizing or reducing agents. Thus, this possibility cannot be ruled out.

Editorial

For figure 1c, distance should be distance to initial entry.

Can the authors supply a better western blot figure 3C? The way the gel ran and the lane difference make it appear as if GluN1 and GAPDH are from different gels.

Concerning the possibility that IH is acting on unitary conductance of the NMDAr or down regulating the receptor. The authors state: This possibility would not be resolved through further electrophysiological investigation alone. This sentence should be removed or modified. The possibility could be resolved with patch-clamp electrophysiological studies.

These findings indicated that IH-dependent HIF1a signaling suppresses NMDAr expression within the hippocampus, that would contribute to a reduction of NMDAr conductance within individual CA1 neurons. Change to . . . hippocampus, which could . . . The word change, from would to could, is needed since it is not clear that the NMDAr expression represents synaptic receptors.

R2:

This study examines the mechanisms and the role of the transcription factor, hypoxia-inducible factor 1a (HIF1a), in cognitive and synaptic function impairment caused by sleep apnea. The rationale is that sleep apnea induces intermittent hypoxia, which is a major mediator of pathophysiology including decline in cognitive and synaptic function. Here, the authors therefore use the Barnes maze that examines hippocampal-dependent spatial memory, brain slice electrophysiology to measure synaptic plasticity in wild type and heterozygous Hif1a mice exposed to intermittent hypoxia and treated with antioxidant, MnTMPyP. In wild type mice, intermittent hypoxia-induced impaired spatial memory was associated with a decline in NMDA receptor dependent LTP and an increase in nuclear factor HIF1a within hippocampus. Intermittent hypoxia-induced HIF1a signaling was also associated with augmented expression of NADPH oxidase 4 and downregulation of GluN1 expression. The intermittent hypoxia-induced effects and HIF1a signaling were not observed in heterozygous for Hif1a or wild type mice treated with MnTMPyP.

The behavioral and physiological results are interesting and the data are surely novel and make a significant contribution. Suggestions for clarification and improvement.

I would raise the point that of the relationship between the title and the content of the manuscript. The title reads “A HIF1a-dependent pro-oxidant state disrupts synaptic plasticity and impairs spatial memory in a model of sleep apnea” and I was building up to it, to this idea of that “sleep apnea will be induced in these animals at some point before behavioral, synaptic, and other measurements. However, I do not think sleep apnea was induced, rather animals were exposed to intermittent hypoxia, and inclusion of heterozygous Hif1a and treatment with antioxidant. I would suggest a change in the title of the manuscript, something like “A HIF1a-dependent pro-oxidant state disrupts synaptic plasticity and impairs spatial memory in mice”. If the authors thinks that heterozygous Hif1a mouse is a model of sleep apnea. In that case, please provide a discussion in the introduction/methods.

I am very enthusiastic for the authors to use P50 to P80 mice for delineating the role of HiF1a signaling in intermittent hypoxia induced-alterations in spatial memory and synaptic plasticity. Considering that, these are very young animals and might be passing just through their developing stage (P50), I am wondering how these findings will be interpreted in relation to adult animals. However, if for whatever reasons of available time/availability of adult animals/commitments, and you are not in a position currently to employ advanced age animals, then I would suggest please provide a short discussion in that perspective.

NMDA receptor is a heterotetramer, consisting of GluN1, an obligatory subunit and GluN2A-D subunit as optional ones. In my opinion, inclusion of expression profile of GluN2A/GluN2B in the current study will definitely make it more interesting and overall status of subunit expression profile during intermittent hypoxia.

Minor points

1) Expand and abbreviate NMDAr (line 43), N-methyl-D-aspartate receptor (NMDAr).

2) Abbreviate “the field excitatory postsynaptic potential” at first use (fEPSP) (line 97).

3) Expand PCNA at first use (line 119), Proliferating Cell Nuclear Antigen.

4) Expand and abbreviate long-term potentiation (LTP) at first use (line 109).

5) Delete “changed” from “protein carbonyl content was elevated in IH10 yet unchanged changed in homogenates” (line 231).

6) Same at line 236, “elevated in IH10 yet unchanged changed in homogenates from either 0-HIF1a+/- or 10-HIF1a+/"

7) Expand and abbreviate DMOG at first use (line 271) in case you are using the same again, Dimethyloxalylglycine (DMOG).

8) Be consistent with the use of MnTMPyP, in line 286, it is written MnTMyPyP.

9) Add an arrow for HFS/TBS, indicating start of stimulation paradigm for induction of LTP, in figure 2.

10) Both side of hippocampi from a mouse were included for Western blot and carbonyl protein analysis or hippocampus was isolated into ventral hippocampus, CA3, CA1, and DG, and region-specific tissues were pooled together? Please provide details in method section.

11) Change reactive oxygen species to ROS (line 277).

12) It might be worth including citations by Bodhinathan et al, 2010, Journal of Neuroscience; Haxaire et al 2012, Aging Cell; Kumar and Foster 2013, Journal of Neuroscience, which would enhance the author’s point that modulation of oxidative state can influence NMDA receptor synaptic function and spatial memory (line 287).

13) Please use expanded version of IP-3 receptor at first use, Inositol 1,4,5-trisphosphate (IP3) receptor (line 292).

14) Editorials, “approach to enhancing HIF1a” approach to enhance (line 272); “by which sleep apnea may lower of the threshold for cognitive impairment", delete of (line 301-2)?

## Author Response

We would like to thank both of the reviewers for their thoughtful input. Based on their specific comments, we added additional experiments revised the manuscript, which has improved the manuscript. Our response to specific issues raised are described below.

R1:

The authors did a nice job and have a fairly complete story. Below are suggestions to improve the manuscript. Major concerns relate to needed comparisons between WT and Hif1a+/- mice. In addition, I have concerns about the proposed link between impaired LTP, NMDAr function, and NMDAr expression.

Our Response: We would like to thank Reviewer 1 for the assessment of our study. Our detailed response to the specific issues raised are stated below.

Methods

For measures of nuclear Hif1a, when was the tissue harvested relative to treatment?

Our Response: Tissue for HIF1a, NOX4, and NR1 was harvested approximately 12 to 16 hours following the ending of the IH protocol on the tenth day of exposure. We have revised the manuscript to reflect this, see lines 163 to 165.

Barnes Maze began on the seventh day of IH10 exposure with respective controls run at the same time. Was the testing performed immediately after the treatment on day 7?

Our Response: The Barnes maze protocol was started prior to the beginning of IH exposure. Barnes Maze began on the seventh day of IH10 exposure with respective controls run at the same time. In IH mice, all training trials and the probe trial were conducted prior to IH exposure on days 7 to 10. We have revised the manuscript to reflect this, see lines 113 to 115.

Define primary latency to exit zone versus initial latency. Is there a behavioral difference in WT and Hif1a+/-? It appears there may be a difference in learning for the controls (Fig 1c vs Fig1f).

Our Response: We apologize for the confusion. Primary latency and latency to initial entry are synonymous in meaning. To reduce confusion, we have revised the text and replaced primary latency with latency to initial entry. Total latency used in training was defined as the elapsed time from the start of the trial to when subject entered the exit. If the mouse was unable to locate the exit by six minutes, they were gently guided to the exit and total latency for the trial was reported as 360 sec.

During the first training trial, many of the mice from 0-HIF1a+/- needed to be guided to the exit or had a very large total latency. However, by the end of third training trial, the group exhibited evidence for learning the location of the exit, as evidenced by the smaller total latency values.

Both distance and latency to initial entry into the exit zone were similar to control (Fig1C. vs. Fig1G). There may have been some confusion when comparing entry probability for 0-HIF1a+/- to control (Fig1D vs. Fig1H). By eye 0-HIF1a+/- mice appear to possess a smaller entry probability than control, when looking at Fig 1D and Fig 1H. However, in the original manuscript the scales on the quantified data (in 1D and 1H) were different. We have revised the Figure 1 to put Fig 1D and 1H on the same scale to remove the illusion for a potential difference and reduce confusion for the reader. Thank you for bring this to our attention.

One assumption is that the LTP in Hif1a+/- mice is due to NMDAr; however, this was not shown (i.e. include AP5). This data needs to be included.

Our Response: We completely agree with the reviewer that we had assumed TBS would evoke NMDAr-dependent LTP in hippocampal slices from HIF1a+/- mice. We have now included an AP5 group in the 10-gHIF1a to illustrate that TBS-LTP was indeed NMDAr-dependent in the HIF1a+/- mice. We have revised the manuscript to reflect this, see lines 256 to 261 and Figure 2D light green symbols.

It is difficult to measure decay time of NMDAr synaptic responses in extracellular recording and there are several problems in the current study.

Our Response: Based on the reviewer’s comments we have chosen to replace the analysis of decay time of the fEPSP with amplitude comparisons as suggested by the reviewer. We have revised the manuscript to reflect this, see lines 262 to 276 and Figure 3A-3B.

1) One problem is the peak amplitudes are vastly different before and after application of AP5 in control mice. Was there a difference in the input/output relationship (AMPAr or NMDAr) between control and Hif1a+/- mice? Was there a difference in input/output curves before and after AP5? At the very least, was there a difference in the maximally evoked fEPSP across mouse strains or due to AP5 application? It appears that AP5 results in a large decrease in the amplitude for the WT control slices (Fig3A). This is not observed for IH10 or Hif1a+/- mice, which suggests fewer NMDARs are at the synapse following IH in WT mice and that synaptic NMDAr levels are low in Hif1a+/- mice regardless of treatment. The question of amplitude differences is also important for interpretation of western blot studies of subunit expression and possible synaptic loss (see Discussion comments).

Our Response: Comparison of maximum amplitudes (evoked by a saturating stimulus current, 700microA) are now replace the decay time measurements of the fEPSP. We did not observe differences between the maximum fEPSP amplitudes generated between in either aCSF or Mg2+-free aCSF. We have revised the manuscript to reflect this, see lines 262 to 276 and Figure 3A and 3B.

In summary, we did not observe differences in the change of fEPSP amplitude in Mg2+-free aCSF from aCSF (Figure 3B) but observed that the reduction of the fEPSP by AP5 was smaller to that of control (Figure 3B). Comparisons of the change in fEPSP of 0-HIF1a+/- and 10-HIF1a+/- to control revealed similar sensitivity to AP5.

2) Second, in order to measure the true decay, the responses should be normalized to the peak (i.e. peak = 100%). Thus, the peaks in each panel should lineup on the horizontal plane and decay differences would then be evident. In figure 3, the decay is contaminated by differences in the peak.

Our Response: We have replaced this analysis with amplitude analyses to be make this less confusing for the reader. (See above)

Discussion

Missing from the discussion is the mechanism for the decrease in LTP and apparent decrease in the NMADr response. The authors state: our findings indicate that IH reduces receptor expression causing targeted loss of NMDAr-dependent LTP through a HIF1a mediated pro-oxidant mechanism. Implicit in this statement is the assumption that the decrease in LTP is due to a decrease in NMDAr at the synapse.

Our Response: To address the reviewer’s concerns, we have made several revisions. Please see below.

First, the authors should discuss whether the reduction in NMDAr expression represents synapse loss, a decreased level of NMDAr at the synapse in the absence of synaptic loss, or loss from intracellular or extrasynaptic compartments. In this case, input/output curves of the fEPSP would help. If the AMPAr component of transmission is not altered, this would suggest no loss of synapses, but does not rule out redox state changes in NMDAr function. If the input/output curve was also decreased, it is possible that there is synaptic loss. In addition, it would have been helpful if the authors had a measure of synaptic number (e.g. PSD95 western blots).

Our Response: We have revised the discussion to reflect many of the reviewer’s perspective as we agree. See the revised discussion (Lines 340 to 349. We have also included western blot analysis of PSD-95. We did not find differences in PSD-95 expression.

Second, the authors did not test for a decrease in NMDAr function due to redox state as reported by the Lipton and Foster groups. In this case, the authors would have needed to determine the effects of oxidizing or reducing agents. Thus, this possibility cannot be ruled out.

Our Response: While we did not test for the potential role redox modulation here, we recognize this possibility in the discussion. See the revised discussion (Lines 361 to 369).

Editorial

For figure 1c, distance should be distance to initial entry.

Our Response: We change distance traveled for distance to initial entry

Can the authors supply a better western blot figure 3C? The way the gel ran and the lane difference make it appear as if GluN1 and GAPDH are from different gels.

Our Response: We changed the representative blot for Figure 3C.

Concerning the possibility that IH is acting on unitary conductance of the NMDAr or down regulating the receptor. The authors state: This possibility would not be resolved through further electrophysiological investigation alone. This sentence should be removed or modified. The possibility could be resolved with patch-clamp electrophysiological studies.

Our Response: We agree with this comment and have revised.

These findings indicated that IH-dependent HIF1a signaling suppresses NMDAr expression within the hippocampus, that would contribute to a reduction of NMDAr conductance within individual CA1 neurons. Change to . . . hippocampus, which could . . . The word change, from would to could, is needed since it is not clear that the NMDAr expression represents synaptic receptors.

Our Response: We have revised this passage.

R2:

This study examines the mechanisms and the role of the transcription factor, hypoxia-inducible factor 1a (HIF1a), in cognitive and synaptic function impairment caused by sleep apnea. The rationale is that sleep apnea induces intermittent hypoxia, which is a major mediator of pathophysiology including decline in cognitive and synaptic function. Here, the authors therefore use the Barnes maze that examines hippocampal-dependent spatial memory, brain slice electrophysiology to measure synaptic plasticity in wild type and heterozygous Hif1a mice exposed to intermittent hypoxia and treated with antioxidant, MnTMPyP. In wild type mice, intermittent hypoxia-induced impaired spatial memory was associated with a decline in NMDA receptor dependent LTP and an increase in nuclear factor HIF1a within hippocampus. Intermittent hypoxia-induced HIF1a signaling was also associated with augmented expression of NADPH oxidase 4 and downregulation of GluN1 expression. The intermittent hypoxia-induced effects and HIF1a signaling were not observed in heterozygous for Hif1a or wild type mice treated with MnTMPyP.

The behavioral and physiological results are interesting and the data are surely novel and make a significant contribution. Suggestions for clarification and improvement.

Our Response: We would thank the reviewer for their recognition of the importance of this study and the suggestions for clarification and improvement. Below we have addressed your specific comments.

I would raise the point that of the relationship between the title and the content of the manuscript. The title reads “A HIF1a-dependent pro-oxidant state disrupts synaptic plasticity and impairs spatial memory in a model of sleep apnea” and I was building up to it, to this idea of that “sleep apnea will be induced in these animals at some point before behavioral, synaptic, and other measurements. However, I do not think sleep apnea was induced, rather animals were exposed to intermittent hypoxia, and inclusion of heterozygous Hif1a and treatment with antioxidant. I would suggest a change in the title of the manuscript, something like “A HIF1a-dependent pro-oxidant state disrupts synaptic plasticity and impairs spatial memory in mice”. If the authors thinks that heterozygous Hif1a mouse is a model of sleep apnea. In that case, please provide a discussion in the introduction/methods.

Our Response: The protocol of intermittent hypoxia used here is well-accepted to experimental model of sleep apnea. We originally choose to avoid confusion about the use of intermittent hypoxia, as several other paradigms of intermittent hypoxia are not meant to model sleep apnea (e.g., Mitchell GS). However, we can see how that title may be misleading. As such we have revised the title to now state:

A HIF1a-dependent pro-oxidant state disrupts synaptic plasticity and impairs spatial memory in response to intermittent hypoxia.

I am very enthusiastic for the authors to use P50 to P80 mice for delineating the role of HiF1a signaling in intermittent hypoxia induced-alterations in spatial memory and synaptic plasticity. Considering that, these are very young animals and might be passing just through their developing stage (P50), I am wondering how these findings will be interpreted in relation to adult animals. However, if for whatever reasons of available time/availability of adult animals/commitments, and you are not in a position currently to employ advanced age animals, then I would suggest please provide a short discussion in that perspective.

Our Response: We have not revised the manuscript to provide a limited discussion about our perspective of using younger versus older animals (See Lines 370 to 375). As discussed, we find it interesting that IH seems to mimic the change from NMDA receptor dependent to NMDA receptor independent synaptic plasticity observed with aging. We also stress the importance of examining how IH may affect aged animals.

NMDA receptor is a heterotetramer, consisting of GluN1, an obligatory subunit and GluN2A-D subunit as optional ones. In my opinion, inclusion of expression profile of GluN2A/GluN2B in the current study will definitely make it more interesting and overall status of subunit expression profile during intermittent hypoxia.

Our Response: These experiments would enhance our understanding of what is occurring in response to IH among WT animals. As such, we were working on this issue. However, we have not had the opportunity to finish these experiments before laboratory closure due to COVID-19. We ask that the reviewer and editor consider our situation. We do not feel that identifying how NR2A-D subunits are affected by IH should prevent our work from being published at this time. We plan to address this in a future study as soon as we return. Thank you for your consideration in this matter.

Minor Points

1) Expand and abbreviate NMDAr (line 43), N-methyl-D-aspartate receptor (NMDAr). Our Response: We revised as suggested. See line 82

2) Abbreviate “the field excitatory postsynaptic potential” at first use (fEPSP) (line 97). Our Response: We revised as suggested. See line 142.

3) Expand PCNA at first use (line 119), Proliferating Cell Nuclear Antigen.

Our Response: We revised as suggested. See line 166 to 167.

4) Expand and abbreviate long-term potentiation (LTP) at first use (line 109).

Our Response: We revised as suggested. See line 45.

5) Delete “changed” from “protein carbonyl content was elevated in IH10 yet unchanged changed in homogenates” (line 231).

Our Response: We revised as requested.

6) Same at line 236, “elevated in IH10 yet unchanged changed in homogenates from either 0-HIF1a+/- or 10-HIF1a+/”:

Our Response: We revised as requested.

7) Expand and abbreviate DMOG at first use (line 271) in case you are using the same again, Dimethyloxalylglycine (DMOG).

Our Response: We revised as suggested. See line 329.

8) Be consistent with the use of MnTMPyP, in line 286, it is written MnTMyPyP.

Our Response: Thank you for seeing this. We have reviewed the document for inconsistencies.

9) Add an arrow for HFS/TBS, indicating start of stimulation paradigm for induction of LTP, in figure 2.

Our Response: We revised as requested

10) Both side of hippocampi from a mouse were included for Western blot and carbonyl protein analysis or hippocampus was isolated into ventral hippocampus, CA3, CA1, and DG, and region-specific tissues were pooled together? Please provide details in method section.

Our Response: We did not discriminate between ventral or dorsal hippocampus or left/right. For more details in methodology. See line 163-165.

11) Change reactive oxygen species to ROS (line 277).

Our Response: We revised as suggested. See line 352.

12) It might be worth including citations by Bodhinathan et al, 2010, Journal of Neuroscience; Haxaire et al 2012, Aging Cell; Kumar and Foster 2013, Journal of Neuroscience, which would enhance the author’s point that modulation of oxidative state can influence NMDA receptor synaptic function and spatial memory (line 287).

Our Response: Thank you for suggesting these references. We have now included Bodhinathan et al (2010) and Kumar and Foster (2013) in the discussion as they seemed to be the most relevant to our work. See line (Lines 353 to 359).

13) Please use expanded version of IP-3 receptor at first use, Inositol 1,4,5-trisphosphate (IP3) receptor (line 292).

Our Response: We changed. See line 363.

14) Editorials, “approach to enhancing HIF1a” approach to enhance (line 272); “by which sleep apnea may lower of the threshold for cognitive impairment", delete of (line 301-2)? Our Response: This is no longer present in our revision. Thank you.

## References

[B1] Bodhinathan K, Kumar A, Foster TC (2010) Intracellular redox state alters NMDA receptor response during aging through Ca2+/calmodulin-dependent protein kinase II. J Neurosci 30:1914–1924. 10.1523/JNEUROSCI.5485-09.2010 20130200 PMC2853968

[B2] Boric K, Muñoz P, Gallagher M, Kirkwood A (2008) Potential adaptive function for altered long-term potentiation mechanisms in aging hippocampus. J Neurosci 28:8034–8039. 10.1523/JNEUROSCI.2036-08.2008 18685028 PMC2615232

[B3] Cha J, Zea-Hernandez JA, Sin S, Graw-Panzer K, Shifteh K, Isasi CR, Wagshul ME, Moran EE, Posner J, Zimmerman ME, Arens R (2017) The effects of obstructive sleep apnea syndrome on the dentate gyrus and learning and memory in children. J Neurosci 37:4280–4288. 10.1523/JNEUROSCI.3583-16.2017 28320844 PMC5413176

[B4] Choi YB, Lipton SA (2000) Redox modulation of the NMDA receptor. Cell Mol Life Sci 57:1535–1541. 10.1007/pl00000638 11092448 PMC11147125

[B5] Chou YT, Zhan G, Zhu Y, Fenik P, Panossian L, Li Y, Zhang J, Veasey S (2013) C/EBP homologous binding protein (CHOP) underlies neural injury in sleep apnea model. Sleep 36:481–492. 10.5665/sleep.2528 23564995 PMC3612250

[B6] Christakis DA, Ramirez JS, Ramirez JM (2012) Overstimulation of newborn mice leads to behavioral differences and deficits in cognitive performance. Sci Rep 2:546. 10.1038/srep00546 22855702 PMC3409385

[B7] Devita M, Montemurro S, Ramponi S, Marvisi M, Villani D, Raimondi MC, Rusconi ML, Mondini S (2017a) Obstructive sleep apnea and its controversial effects on cognition. J Clin Exp Neuropsychol 39:659–669. 10.1080/13803395.2016.1253668 27845600

[B8] Devita M, Montemurro S, Zangrossi A, Ramponi S, Marvisi M, Villani D, Raimondi MC, Merlo P, Rusconi ML, Mondini S (2017b) Cognitive and motor reaction times in obstructive sleep apnea syndrome: a study based on computerized measures. Brain Cogn 117:26–32. 10.1016/j.bandc.2017.07.002 28700954

[B9] Diebold I, Petry A, Hess J, Görlach A (2010) The NADPH oxidase subunit NOX4 is a new target gene of the hypoxia-inducible factor-1. Mol Biol Cell 21:2087–2096. 10.1091/mbc.e09-12-1003 20427574 PMC2883952

[B10] Foster TC, Kyritsopoulos C, Kumar A (2017) Central role for NMDA receptors in redox mediated impairment of synaptic function during aging and Alzheimer’s disease. Behav Brain Res 322:223–232. 10.1016/j.bbr.2016.05.012 27180169

[B11] Gildeh N, Drakatos P, Higgins S, Rosenzweig I, Kent BD (2016) Emerging co-morbidities of obstructive sleep apnea: cognition, kidney disease, and cancer. J Thorac Dis 8:E901–E917. 10.21037/jtd.2016.09.23 27747026 PMC5059346

[B12] Goldbart A, Cheng ZJ, Brittian KR, Gozal D (2003) Intermittent hypoxia induces time-dependent changes in the protein kinase B signaling pathway in the hippocampal CA1 region of the rat. Neurobiol Dis 14:440–446. 10.1016/j.nbd.2003.08.004 14678760

[B13] Gozal D, Daniel JM, Dohanich GP (2001) Behavioral and anatomical correlates of chronic episodic hypoxia during sleep in the rat. J Neurosci 21:2442–2450. 10.1523/JNEUROSCI.21-07-02442.2001 11264318 PMC6762394

[B14] Gozal D, Row BW, Gozal E, Kheirandish L, Neville JJ, Brittian KR, Sachleben LR Jr, Guo SZ (2003) Temporal aspects of spatial task performance during intermittent hypoxia in the rat: evidence for neurogenesis. Eur J Neurosci 18:2335–2342. 10.1046/j.1460-9568.2003.02947.x 14622195

[B15] Guidi M, Kumar A, Foster TC (2015) Impaired attention and synaptic senescence of the prefrontal cortex involves redox regulation of NMDA receptors. J Neurosci 35:3966–3977. 10.1523/JNEUROSCI.3523-14.2015 25740525 PMC4348191

[B16] Iyer NV, Kotch LE, Agani F, Leung SW, Laughner E, Wenger RH, Gassmann M, Gearhart JD, Lawler AM, Yu AY, Semenza GL (1998) Cellular and developmental control of O2 homeostasis by hypoxia-inducible factor 1 alpha. Genes Dev 12:149–162. 10.1101/gad.12.2.149 9436976 PMC316445

[B17] Khuu MA, Pagan CM, Nallamothu T, Hevner RF, Hodge RD, Ramirez JM, Garcia AJ 3rd (2019) Intermittent hypoxia disrupts adult neurogenesis and synaptic plasticity in the dentate gyrus. J Neurosci 39:1320–1331. 10.1523/JNEUROSCI.1359-18.2018 30587544 PMC6381238

[B18] Kumar A, Foster TC (2013) Linking redox regulation of NMDAR synaptic function to cognitive decline during aging. J Neurosci 33:15710–15715. 10.1523/JNEUROSCI.2176-13.2013 24089479 PMC3787496

[B19] Leng Y, McEvoy CT, Allen IE, Yaffe K (2017) Association of sleep-disordered breathing with cognitive function and risk of cognitive impairment: a systematic review and meta-analysis. JAMA Neurol 74:1237–1245. 10.1001/jamaneurol.2017.2180 28846764 PMC5710301

[B20] Lipton SA, Choi YB, Takahashi H, Zhang D, Li W, Godzik A, Bankston LA (2002) Cysteine regulation of protein function–as exemplified by NMDA-receptor modulation. Trends Neurosci 25:474–480. 10.1016/s0166-2236(02)02245-2 12183209

[B21] Macey PM, Prasad JP, Ogren JA, Moiyadi AS, Aysola RS, Kumar R, Yan-Go FL, Woo MA, Albert Thomas M, Harper RM (2018) Sex-specific hippocampus volume changes in obstructive sleep apnea. Neuroimage Clin 20:305–317. 10.1016/j.nicl.2018.07.027 30101062 PMC6083433

[B22] Nair D, Dayyat EA, Zhang SX, Wang Y, Gozal D (2011) Intermittent hypoxia-induced cognitive deficits are mediated by NADPH oxidase activity in a murine model of sleep apnea. PLoS One 6:e19847. 10.1371/journal.pone.0019847 21625437 PMC3100309

[B23] Payne RS, Goldbart A, Gozal D, Schurr A (2004) Effect of intermittent hypoxia on long-term potentiation in rat hippocampal slices. Brain Res 1029:195–199. 10.1016/j.brainres.2004.09.045 15542074

[B24] Peng YJ, Prabhakar NR (2003) Reactive oxygen species in the plasticity of respiratory behavior elicited by chronic intermittent hypoxia. J Appl Physiol 94:2342–2349. 10.1152/japplphysiol.00613.2002 12533494

[B25] Peng YJ, Yuan G, Ramakrishnan D, Sharma SD, Bosch-Marce M, Kumar GK, Semenza GL, Prabhakar NR (2006) Heterozygous HIF-1alpha deficiency impairs carotid body-mediated systemic responses and reactive oxygen species generation in mice exposed to intermittent hypoxia. J Physiol 577:705–716. 10.1113/jphysiol.2006.114033 16973705 PMC1890436

[B26] Prabhakar NR, Semenza GL (2012) Adaptive and maladaptive cardiorespiratory responses to continuous and intermittent hypoxia mediated by hypoxia-inducible factors 1 and 2. Physiol Rev 92:967–1003. 10.1152/physrev.00030.2011 22811423 PMC3893888

[B27] Robillard JM, Gordon GR, Choi HB, Christie BR, MacVicar BA (2011) Glutathione restores the mechanism of synaptic plasticity in aged mice to that of the adult. PLoS One 6:e20676. 10.1371/journal.pone.0020676 21655192 PMC3105108

[B28] Row BW, Kheirandish L, Neville JJ, Gozal D (2002) Impaired spatial learning and hyperactivity in developing rats exposed to intermittent hypoxia. Pediatr Res 52:449–453. 10.1203/00006450-200209000-00024 12193683

[B29] Semenza GL, Prabhakar NR (2015) Neural regulation of hypoxia-inducible factors and redox state drives the pathogenesis of hypertension in a rodent model of sleep apnea. J Appl Physiol 119:1152–1156. 10.1152/japplphysiol.00162.2015 25953833 PMC4816415

[B30] Sforza E, Celle S, Saint-Martin M, Barthélémy JC, Roche F (2016) Hippocampus volume and subjective sleepiness in older people with sleep-disordered breathing: a preliminary report. J Sleep Res 25:190–193. 10.1111/jsr.12367 26662175

[B31] Song X, Roy B, Kang DW, Aysola RS, Macey PM, Woo MA, Yan-Go FL, Harper RM, Kumar R (2018) Altered resting-state hippocampal and caudate functional networks in patients with obstructive sleep apnea. Brain Behav 8:e00994. 10.1002/brb3.994 29749715 PMC5991585

[B32] Varga AW, Kishi A, Mantua J, Lim J, Koushyk V, Leibert DP, Osorio RS, Rapoport DM, Ayappa I (2014) Apnea-induced rapid eye movement sleep disruption impairs human spatial navigational memory. J Neurosci 34:14571–14577. 10.1523/JNEUROSCI.3220-14.2014 25355211 PMC4212062

[B33] Wall AM, Corcoran AE, O'Halloran KD, O'Connor JJ (2014) Effects of prolyl-hydroxylase inhibition and chronic intermittent hypoxia on synaptic transmission and plasticity in the rat CA1 and dentate gyrus. Neurobiol Dis 62:8–17. 10.1016/j.nbd.2013.08.016 24055213

[B34] Wallace A, Bucks RS (2013) Memory and obstructive sleep apnea: a meta-analysis. Sleep 36:203–220. 10.5665/sleep.2374 23372268 PMC3543053

[B35] Xie H, Leung KL, Chen L, Chan YS, Ng PC, Fok TF, Wing YK, Ke Y, Li AM, Yung WH (2010) Brain-derived neurotrophic factor rescues and prevents chronic intermittent hypoxia-induced impairment of hippocampal long-term synaptic plasticity. Neurobiol Dis 40:155–162. 10.1016/j.nbd.2010.05.020 20553872

[B36] Yuan G, Nanduri J, Khan S, Semenza GL, Prabhakar NR (2008) Induction of HIF-1alpha expression by intermittent hypoxia: involvement of NADPH oxidase, Ca2+ signaling, prolyl hydroxylases, and mTOR. J Cell Physiol 217:674–685. 10.1002/jcp.21537 18651560 PMC2696817

[B37] Zhang SX, Wang Y, Gozal D (2012) Pathological consequences of intermittent hypoxia in the central nervous system. Compr Physiol 2:1767–1777. 10.1002/cphy.c100060 23723023

[B38] Zhdanov AV, Okkelman IA, Collins FW, Melgar S, Papkovsky DB (2015) A novel effect of DMOG on cell metabolism: direct inhibition of mitochondrial function precedes HIF target gene expression. Biochim Biophys Acta 1847:1254–1266. 10.1016/j.bbabio.2015.06.016 26143176

